# Microfilament‐Myosin II Regulates the Differentiation of Multinucleated Cysts into Oocytes and Influences Oocyte Developmental Potential in Mice

**DOI:** 10.1002/advs.202500358

**Published:** 2025-09-17

**Authors:** Rui Xu, Menghao Pan, Zhi Zheng, Yaju Tang, Yutong Yan, Sihai Lu, Sha Peng, Baohua Ma

**Affiliations:** ^1^ College of Veterinary Medicine Northwest A&F University Yangling Shaanxi 712100 China

**Keywords:** mice, microfilament, multinucleated cysts, myosin II, oocyte differentiation

## Abstract

Primary oocyte differentiation in females is a complex and selective process; however, its regulatory mechanisms remain poorly understood. In this study, multinucleated cysts are identified as precursors for oocyte differentiation. Disruption of microfilament dynamics disrupts the differentiation of multinucleated cysts into oocytes, resulting in reduced oocyte volume, polarity defects, Balbiani body (B‐body) absence, and double‐nucleated oocytes. Proteomics analysis reveals that microfilament depolymerization alters the myosin II isoform expression and decreases myosin II activity. Subcellular localization analysis of myosin II in multinucleated cysts demonstrates that myosin IIA co‐localized with microfilaments in the cortex and cytoplasm; myosin IIB is associated with the Golgi apparatus; and myosin IIC is distributed diffusely in the cytoplasm. Pharmacological inhibition of myosin II activity or specific knockdown of myosin II isoforms leads to oocyte differentiation abnormalities, including reduced oocyte volume, failed B‐body formation, and double‐nucleus phenotypes. Compromised differentiation quality of oocytes has long‐term consequences, with defective oocytes showing impaired developmental progression, elevated apoptosis, and diminished meiotic competence. These defects are evidenced by reduced rates of germinal vesicle breakdown (GVBD) and first polar body (PB1) excretion, mitochondrial dysfunction, reduced Golgi and endoplasmic reticulum components, and increased spindle abnormalities. In conclusion, this study identifies multinucleated cysts as precursors for oocyte differentiation, highlighting the importance of microfilament myosin II in oocyte differentiation and its long‐term influence on oocyte differentiation quality.

## Graphical abstract

In this study, we provided new evidence identifying multinucleated cysts as precursors for oocyte differentiation. Within these cysts, organelles migrate and aggregate to form Balbiani body (B‐body) and establish early cellular polarity. Concurrently, excess nuclei are expelled, enabling cyst‐to‐oocyte differentiation. The microfilament‐myosin II plays a crucial role in these processes. Any disruption of these dynamic changes or activities affects the quality of oocyte differentiation. Furthermore, poor oocyte differentiation negatively hinders the subsequent development and maturation of oocytes.

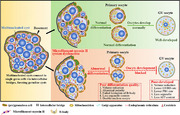

## 1 Introduction

The reproductive lifespan of female mammals is determined by the number of primordial follicles (PFs) in the PF pool, and the formation of PFs is vital to ensure a sufficiently large PF pool. During PF formation, primordial germ cells (PGCs) first differentiate into oocytes in the germline cyst, after which the pre‐granulosa cells migrate into the cyst, leading them to break down and enclose the oocytes to form PFs.^[^ ^1^ ^]^ In many species, the differentiation of oocytes into germline cysts requires organelles and cytoplasm from sister germ cells (also called nurse cells) to enrich their content.^[^ ^1c^ ^]^ In female fetal mice, PGCs migrate to the genital ridge around embryonic days 10.5 (E10.5) and become oogonia. These germ cells undergo several rounds of incomplete mitosis until ∼E14.5, forming germline cysts. At this point, sister germ cells connect to each other through intercellular bridges to transfer organelles and cytoplasm in a non‐synchronized manner. This transport starts from E15.5, peaks at E17.5‐E19.5 (postnatal day 0 [P0]), and gradually reaches an end on postnatal day 2 (P2).^[^ ^1a^ ^]^ Germ cells that have completed mitosis almost simultaneously proceed to enter meiosis at E14.5. Although nurse cells that had begun to transfer their cytoplasm largely follow meiotic expression, meiotic expression in these cells tends to be weaker.^[^ ^2^ ^]^

In oocytes that have completed differentiation, the Balbiani body (B‐body), a highly conserved structure, is observed in the cytoplasm.^[^ ^3^ ^]^ Most of the cytoplasmic components are enriched there and are characterized by abundant organelles, including a ring Golgi apparatus and its internal centrosomes, mitochondria, endoplasmic reticulum (ER), and another germplasm (RNAs and proteins that specify PGCs in early embryos).^[^ ^1a^ ^]^ Therefore, oocytes tend to exhibit pronounced asymmetric morphologies and polarities at this stage.^[^ ^4^ ^]^ The fate of germ cells is not determined randomly; only 20% of these cells will become recipients and ultimately differentiate into oocytes, whereas 80% will contribute cytoplasmic components and undergo death.^[^ ^2^, ^5^ ^]^ Studies in mice have shown that germ cells connected by three or four bridges, namely branching germ cells, are preferentially protected from cell death and accumulate cytoplasm and organelles from sister germ cells to become primary oocytes.^[^ ^6^ ^]^ However, in humans, multinucleated cysts have been proven to be the precursors that differentiate into oocytes. A multinucleated cyst is not a single germ cell but a large cell structure containing 2–4 nuclei, and the multinucleated cyst can also connect to the surrounding single germ cells by intercellular bridges.^[^ ^1d^ ^]^

Intercellular bridges are conserved throughout gametogenesis in several species.^[^ ^6^, ^7^ ^]^ In mammalian germ cells, the intercellular bridge consists of cytoplasmic components and germ cell‐specific factors such as testis‐expressed gene 14 (TEX14).^[^ ^8^ ^]^ TEX14 is an inactive kinase that plays a crucial role in maintaining the stability of intercellular bridges.^[^ ^9^ ^]^ Previous studies have demonstrated the importance of these bridges in the transport of cytoplasmic components. Germ cells with more intercellular bridges are more likely to be shielded from apoptosis and eventually develop into oocytes.^[^ ^6^ ^]^ However, in TEX14‐knockout female mice, the germ cells can connect through syncytia or fragmented membranes when the intercellular bridges are absent, and this process does not result in infertility. These findings suggest that intercellular bridges do not play a pivotal role in germ cell connectivity, the generation of the oocyte pool, or oocyte quality and functionality.^[^ ^10^ ^]^ Thus, alternative methods for enhancing cytoplasmic components that do not rely on intercellular bridges may also be available during oocyte differentiation.

The cytoskeleton is a network of protein fibers located on the inner surface of cell membranes, and is mainly composed of microtubules, microfilaments, and intermediate filaments.^[^ ^11^ ^]^ Its main functions can be summarized as follows: 1) it maintains cell morphology and the locations of various organelles; 2) it is involved in the transport and signal transduction of various organelles and biomacromolecules in the cells; and 3) as the cell's power device, it participates in various cell movements, including cell division and migration.^[^ ^12^ ^]^ The diverse physiological functions of the cytoskeleton are often mediated through coordinated interactions with specialized motor proteins such as kinesin and dynein, which bind to microtubules, and myosin, which binds to microfilaments.^[^ ^13^ ^]^ The important roles of microtubules and their motility proteins in germ cell differentiation have been previously confirmed. Organelle transport occurs along the polarized microtubule cytoskeleton, and culturing of ovaries with low levels of anti‐microtubule drugs has been shown to impair the formation of primary oocytes and Balbiani bodies.^[^ ^1^, ^14^ ^]^ However, the effects of microfilaments and their co‐proteins in oocyte differentiation have not been fully clarified. Intercellular bridges in female *Drosophila* are supported by a robust filamentous actin (F‐actin) cytoskeleton, suggesting that microfilaments serve as the structural basis for intercellular bridges to support intercellular cytoplasmic transport.^[^ ^15^ ^]^ F‐actin tethers nurse cell nuclei to prevent them from entering oocytes before bulk cytoplasmic transfer in *Drosophila* and mouse germline cysts.^[^ ^2^, ^16^ ^]^ The activity of myosin II, a dynamic protein of microfilaments, has been proven to be crucial for regulating the differentiation of *Drosophila* oocytes.^[^ ^17^ ^]^ On the other hand, the movement of organelles, plasma membrane fluidity, and cytoplasmic flow are interconnected with the dynamic changes in microfilaments. For example, the ARP2/3 complex affects cytoplasmic flow, intracellular material transport, and cell migration by regulating microfilament assembly and branching.^[^ ^18^ ^]^ Nevertheless, the exact functions of microfilaments in germ cell differentiation, particularly in cytoplasmic transport, remain to be elucidated.

The potential characteristics that can distinguish the different differentiation fates of germ cells remain unclear, and our understanding of the underlying mechanisms and biological importance of cytoplasmic transport within mouse germ cell cysts remains limited. Intercellular bridges may not be the only way to mediate the enrichment of organelles, because their destruction does not affect reproductive ability. This may indicate a compensatory mechanism that mediates cytoplasmic transport. When oocyte differentiation is obstructed and fails to form a B‐body by disrupting the cytoplasmic transport process, oocytes cannot develop into later‐stage oocytes. Thus, the quality of early oocyte differentiation is a crucial factor in determining the quality of subsequent mature oocytes. Therefore, considering the aforementioned unresolved issues, this study aimed to elucidate the precursors and differentiation patterns of oocytes, examine the potential involvement of the microfilament cytoskeleton in oocyte differentiation, and evaluate the long‐term effects of oocyte differentiation quality on subsequent development. Through our research, we further identified multinucleated cysts as precursors of oocyte differentiation and established the enrichment and polarity of organelles. The multinucleated cysts exited the excess nuclei to form oocytes. Microfilament myosin II plays an essential role in the differentiation of multinucleated cysts into oocytes. Moreover, the quality of oocyte differentiation affects subsequent follicular development and determines the quality of mature oocytes.

## 2 Results

### 2.1 Multinucleated Cysts Serve as the Precursors for Oocyte Differentiation in Fetal Mice

**Figure 1:**
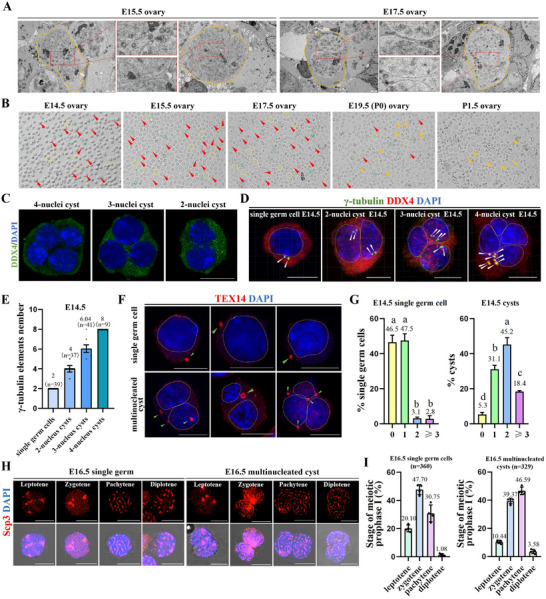
Multinucleated cysts were the precursor for differentiation into oocytes. A) Transmission electron microscopy images of multinucleated cyst in E15.5 and E17.5 ovaries, scale bar = 2 µm. B) Single‐cell suspensions of ovaries form E14.5 to P1.5 fetal or newborn mouse, scale bar = 50 µm. The multinucleated cyst (red arrowhead); single germ cell (yellow dotted circle); somatic cell (green dotted circle); primary oocyte (orange arrowhead). C) Representative images of 4, 3 and 2‐nuclei multinucleated cyst, scale bar = 10 µm. D,E) The number of centrioles in E14.5 single germ cells and multinucleated cysts, scale bar = 10 µm. F,G) The staining and quantitative analysis of intercellular bridges in E14.5 single germ cells and multinucleated cysts, scale bar = 10 µm (E14.5 single germ cell: *n* = 327, E14.5 cysts: *n* = 285). H) Scp3 staining in single germ cells and multinucleated cysts by E16.5, scale bar = 10 µm. I) The ratio of single germ cells and multinucleated cysts in different phases of meiosis. Different letters indicate significant differences (*p* < 0.05).

Through transmission electron microscopy analyses of ovarian sections and digestion of ovaries to single‐cell suspensions, we identified some large multinucleated cells that were different from single germ cells in the ovaries of fetal mice from E14.5∼P1.5, similar to the findings reported in human PF formation.^[^ ^1d^ ^]^ These large multinucleated cells often contained 2–4 closely adjacent germ cell nuclei (**Figure** **1**A–C; Movies S1–S4, Supporting Information), and we defined these cells as multinucleated cysts. As early as E14.5, the centrioles and nuclei of the multinucleated cysts were present in a two‐to‐one ratio (Figure 1D,E). This feature may be an important indicator of the number of germ cells in the earliest formed multinucleated cysts. Next, we evaluated the number of intercellular bridges in multinucleated cysts and single germ cells, since the intercellular bridge may serve as a potential indicator for identifying pre‐oocytes. Germ cells containing more intercellular bridges were preferentially protected and eventually differentiated into oocytes.^[^ ^2^ ^]^ After staining with TEX14 (an intercellular bridge marker), confocal laser scanning showed that intercellular bridges were present on the surface of multinucleated cysts (Figure 1F). Notably, multinucleated cysts contained more bridges than single germ cells (Figure 1G). More bridges allow the multinucleated cysts to receive more cytoplasmic components from single germ cells. All nurse germ cells and pro‐oocytes entered meiosis at E14.5, but nurse cells that had begun cytoplasm transfer largely following the weakly meiotic expression.^[^ ^2^ ^]^ Therefore, we analyzed the meiotic processes and synchrony of multinucleated cysts and single germ cells. The results showed that both multinucleated cysts and single germ cells entered meiosis at E16.5; however, multinucleated cysts often progressed faster than single germ cells (Figure 1H,I). These findings suggest that multinucleated cysts may be a precursor to oocyte differentiation.

### 2.2 Organizational Dynamics of Multinucleated Cysts During Oocyte Differentiation

**Figure 2:**
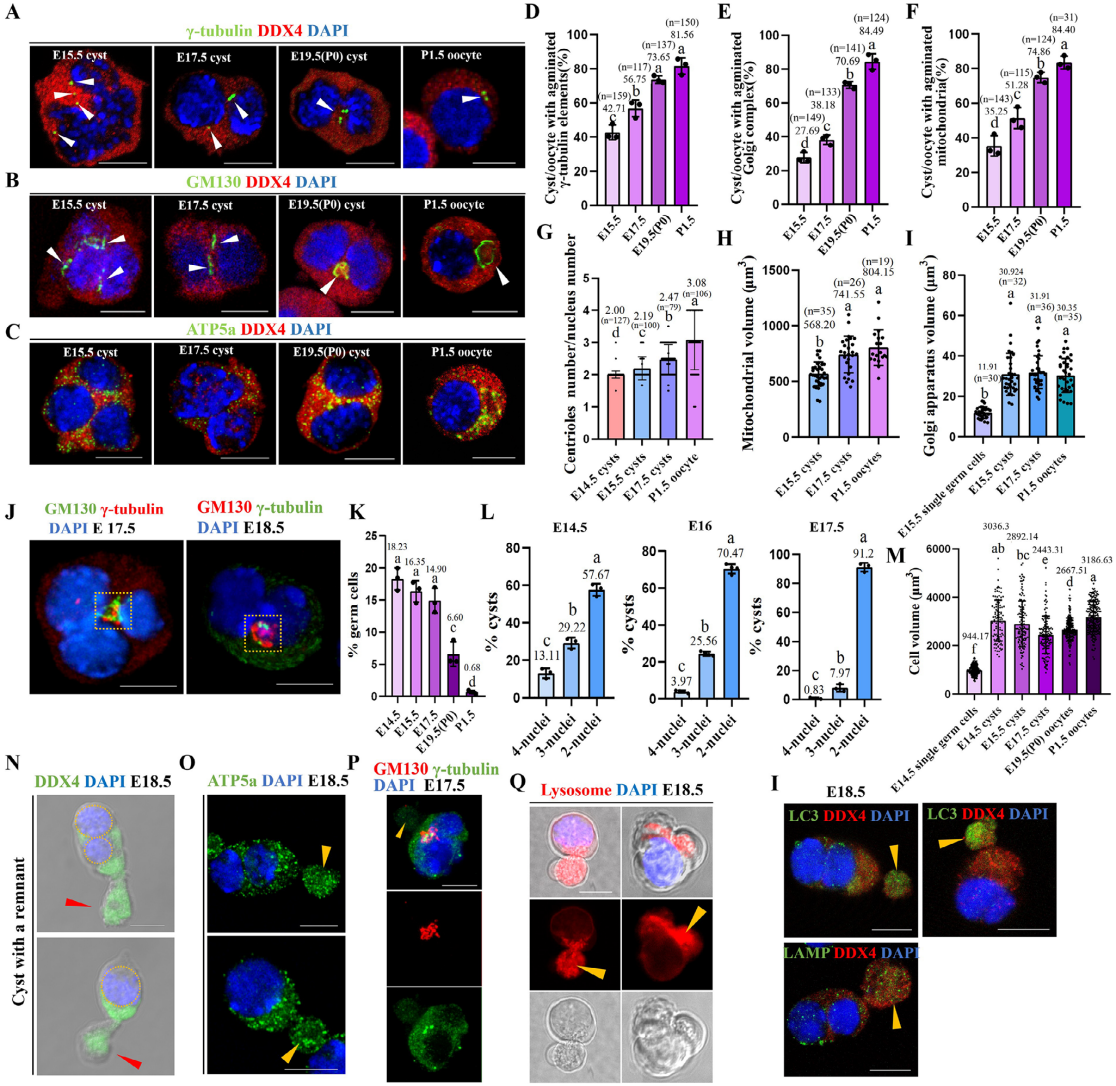
The pattern of multinucleated cysts differentiation into oocytes. A–C) The localization changes of centrioles, Golgi apparatus and mitochondria in multinucleated cysts or oocytes from E15.5 to P1.5. scale bar = 10 µm. D–F) The proportion of agminated centrioles (D), Golgi apparatus (E) and mitochondria (F) in multinucleated cysts or oocytes from E15.5 to P1.5. G–I) The changes in quantity or volume of centrioles, mitochondria and Golgi apparatus in multinucleated cysts or oocytes from E14.5‐P1.5. J) Pre‐B body in multinucleated cyst. scale bar = 10. K) The proportion of multinucleated cysts in total germ cells from E14.5 to P1.5 ovaries. L) The proportion of 4, 3, and 2 nuclei multinucleated cyst in total multinucleated cyst from E14.5‐E17.5 ovaries (E14.5: *n* = 309, E16: *n* = 251, E17.5: *n* = 193). M) The volume of single germ cells, multinucleated cysts and oocytes on different days. N) The multinucleated cyst with a residual body, scale bar = 10 µm. O,P) The residue body contained some mitochondria but not the Golgi apparatus and centrioles, scale bar = 10 µm. Q) The residue body showed strong acidic lysosomal staining. Scale bar = 10 µm. R) The residue was rich in autophagy‐related proteins. Different letters indicate significant differences (*p* < 0.05).

Mouse primary oocytes that completed differentiation built a Balbiani body (B‐body) consisting of a ring Golgi apparatus, centrosomes, a mass of mitochondria, and ER. The B‐body also determined the asymmetric morphology and early polarity of primary oocytes.^[^ ^1^, ^4^ ^]^ However, the mechanisms governing their formation remain to be clarified. In our study, we performed organelle‐specific labeling: GM130 for Golgi apparatus, γ‐tubulin for centrioles, and ATP5a for mitochondria. Surprisingly, we found that the centrioles, Golgi apparatus, and mitochondrial distribution in multinucleated cysts changed from a relatively dispersed pattern in the cytoplasm at E15.5 to a centralized distribution between the nuclei at E19.5(P0) (**Figure** **2**A–C). This spatial reorganization exhibited distinct temporal patterns: the Golgi apparatus formed ring structures in 38.18% of the cysts at E17.5, and this proportion increased to 70.69% by E19.5(P0) (Figure 2E); centriole and mitochondrial clustering occurred earlier, reaching 56.75% and 51.28%, respectively, at E17.5 (Figure 2D,F). Next, we quantified organelle abundance during the cyst‐to‐oocyte transition (E15.5‐P1.5). The results showed that the Golgi apparatus content remained stable across the developmental stages (Figure 2I), indicating that the Golgi apparatus may not be transferred from other germ cells to multinucleated cysts. This was not surprising, as its larger structure most likely limited its transfer between cells via a narrow intercellular bridge. In contrast, the centriole‐to‐nucleus ratios progressively increased, reaching 3.08 centrioles per oocyte by P1.5 (Figure 2G). The mitochondrial content exhibited a stage‐dependent accumulation, peaking in primary oocytes (Figure 2H). Simultaneously, we observed that multinucleated cysts were capable of assembling B‐bodies before their differentiation into primary oocytes (Figure 2J), demonstrating that establishment of polarity and B‐body formation preceded the completion of oocyte differentiation.

We further analyzed the dynamic cyst population shifts. Multinucleated cysts constituted 18.23% of the germ cells at E14.5, but this proportion declined sharply to 0.68% at P1.5 (Figure 2K). Meanwhile, from E14.5‐E17.5, the number of 4‐ and 3‐nuclei multinucleated cysts gradually decreased, whereas the number of 2‐nuclei multinucleated cysts increased (Figure 2L). Concurrently, differentiating oocytes (characterized by B‐body formation and diplotene entry) emerged from E17.5, with cyst volumes progressively approximating those of formed oocytes at E19.5 (Figure 2M).

**Figure 3:**
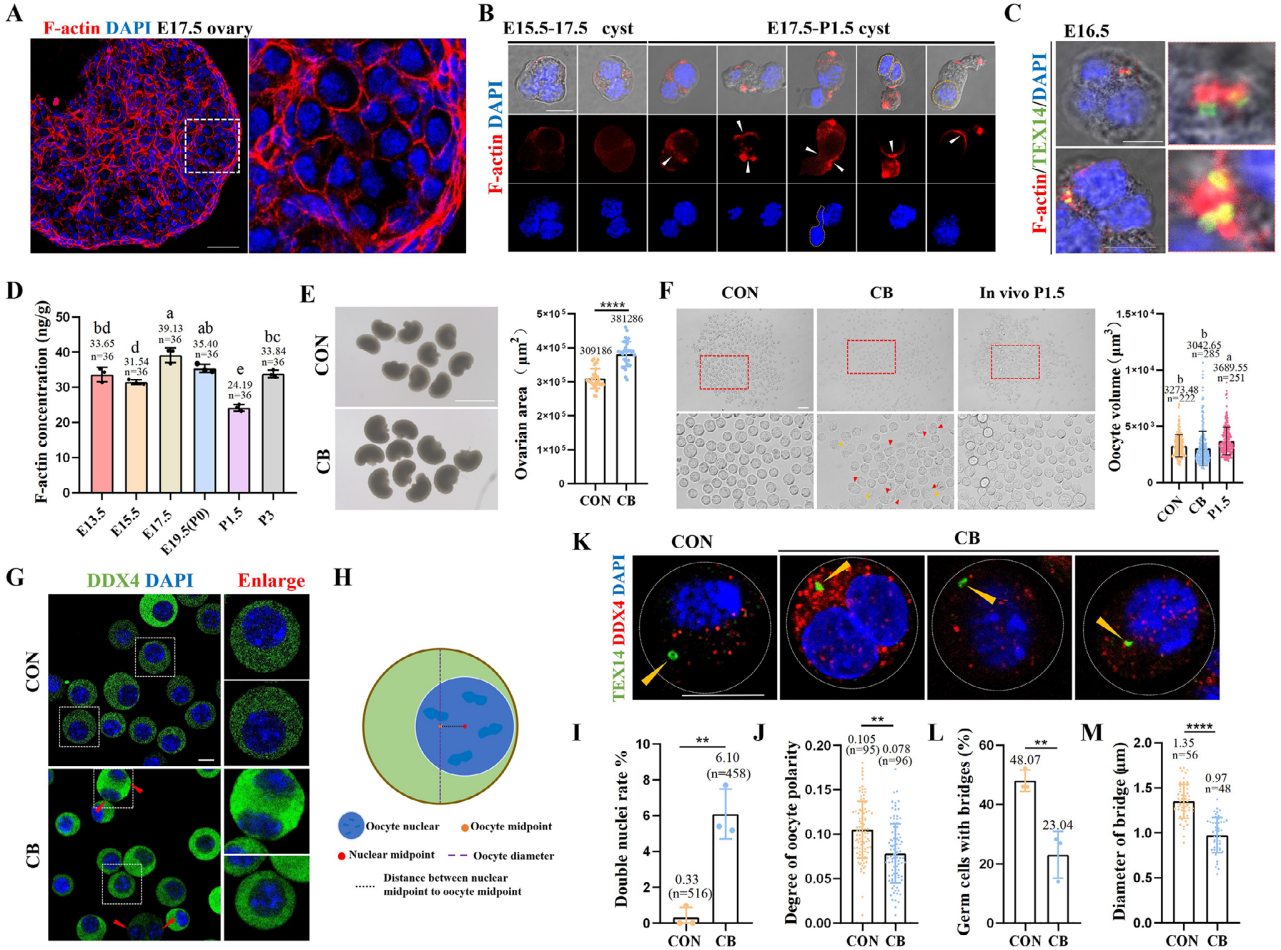
Microfilaments were crucial for the oocyte differentiation. A) Typical pictures of F‐actin staining at E17.5 mouse ovary, scale bar = 100 µm. B) F‐actin staining in multinucleated cyst at different stages, scale bar = 10 µm. C) Co‐staining of TEX14 and F‐actin in multinucleated cyst, scale bar = 10 µm. D) The concentration of F‐actin in ovaries from E14.5‐P3. E) The ovary morphology and volume of E16.5 fetal mouse ovaries after culturing for 4 days in CON and CB treatment groups. F) The morphology and volume of oocytes in CON group, CB treatment group and the P1.5 mouse ovary, scale bar = 100 µm. G) The DDX4 staining of oocytes in CON and CB treatment groups, scale bar = 10 µm. H) The method for calculating oocyte polarity was represented in a schematic diagram. I) The rate of oocytes with double nuclei in CON and CB treatment groups. J) The polarity degree of oocytes in CON group and CB treatment group. K) The morphology of intercellular bridges in CON and CB treatment groups, scale bar = 10 µm. L) The proportion of germ cells with bridges in CON and CB treatment groups (CON: *n* = 266, CB: *n* = 319). M) The intercellular bridge diameter statistics in CON and CB treatment groups. ^*^ and different letters meant *p* < 0.05, ^**^ meant *p* < 0.01, ^****^ meant *p* < 0.0001.

Cytological analysis of ovarian single‐cell suspensions identified residual multinucleated cyst fragments that were positive for the germline marker DDX4 (Figure 2N). These remnants contained mitochondria and excess nuclei but lacked the Golgi apparatus (Figures 2O,P and 3B). Lysosomal tracker staining revealed robust lysosomal accumulation in these remnants (Figure 2Q). LC3 (a marker of autophagy) and LAMP (a lysosomal marker) showed intense staining in the remnants, indicating that these remnants may be degraded through the autophagy pathway (Figure 2I). In summary, cyst‐to‐oocyte transition involves coordinated organelle repositioning, B‐body assembly, and autophagic pruning of non‐essential elements. Collectively, these processes enable the transformation of multinucleated cysts into polarized, functional oocytes.

### 2.3 The Microfilament Cytoskeleton Regulates Oocyte Differentiation

First, the position of microfilaments (F‐actin) was determined during oocyte differentiation. As shown in **Figure** **3**A,B, microfilaments mainly underlie the cortex of germ cells and multinucleated cysts. In multinucleated cysts undergoing residual body expulsion, distinct aggregation of microfilaments was observed between the nuclei, resembling contractile ring formation (Figure 3B). Simultaneously, we observed colocalization between microfilaments and intercellular bridges, and microfilaments were highly expressed at the bases of intercellular bridges (Figure 3C). Quantification analyses showed that F‐actin concentration was significantly higher at E17.5 and E19.5 than at other stages, coinciding with the period of organelle migration and multinucleated cyst reduction (Figure 3D). These findings suggest that microfilaments are involved in the differentiation of multinucleated cysts into oocytes. Subsequently, we treated E16.5 fetal mouse ovaries with 2.5 µg mL^−1^ cytochalasin B (CB), a specific microfilament‐depolymerizing drug, for 4 days in ovarian culture medium. CB treatment significantly reduced the level of F‐actin in germ cells and oocytes after culturing for 1 and 4 d, with a more pronounced effect on germ cells than on somatic cells (Figure S1A–D, Supporting Information). After 4 days of CB treatment, the ovarian volume significantly increased (Figure 3E), whereas oocytes showed morphological abnormalities and reduced volume (Figure 3F). Intriguingly, large‐volume cells resembling multinucleated cysts (red arrowhead) were observed along with instances of incomplete nuclear efflux (yellow arrowhead, Figure 3F). Previous studies have shown that fully differentiated oocytes have clear polarity with the nucleus typically localized on one side, as shown in Figure 3H.^[^ ^4^ ^]^ However, CB treatment disrupted this polarity (Figure 3G,J). Another important finding was the higher incidence of double‐nucleated oocytes (red arrowhead) in the CB group, a phenomenon rarely observed in the CON group (Figure 3G,I).

To assess the dynamic effects of CB, we isolated multinucleated cysts undergoing remnant expulsion and monitored their real‐time changes in CON and CB culture media. As shown in Movies S5 and S6 (Supporting Information), CB‐treated cysts underwent rapid morphological alterations, with retraction of the expelled portion; this phenomenon was not observed in the CON group. Thus, CB treatment reverses the expulsion process, potentially explaining the increased prevalence of multinucleated oocytes in the CB group. Furthermore, microfilament depolymerization disrupted the intercellular bridge, causing disappearance of the annular structure and significantly reducing the bridge diameter (Figure 3K–M). This may hinder the transfer of cytoplasmic components from other germ cells to multinucleated cysts. We also evaluated autophagy and germ cell acidification and found that CB treatment significantly reduced autophagy levels and the number of acidified germ cells (Figure S2A–D, Supporting Information). Collectively, these results indicated that disruption of microfilaments significantly affects the differentiation of multinucleated cysts into oocytes and leads to abnormal oocytes.

### 2.4 Microfilament Depolymerization Reduced the Organelle Content in Oocytes

**Figure 4:**
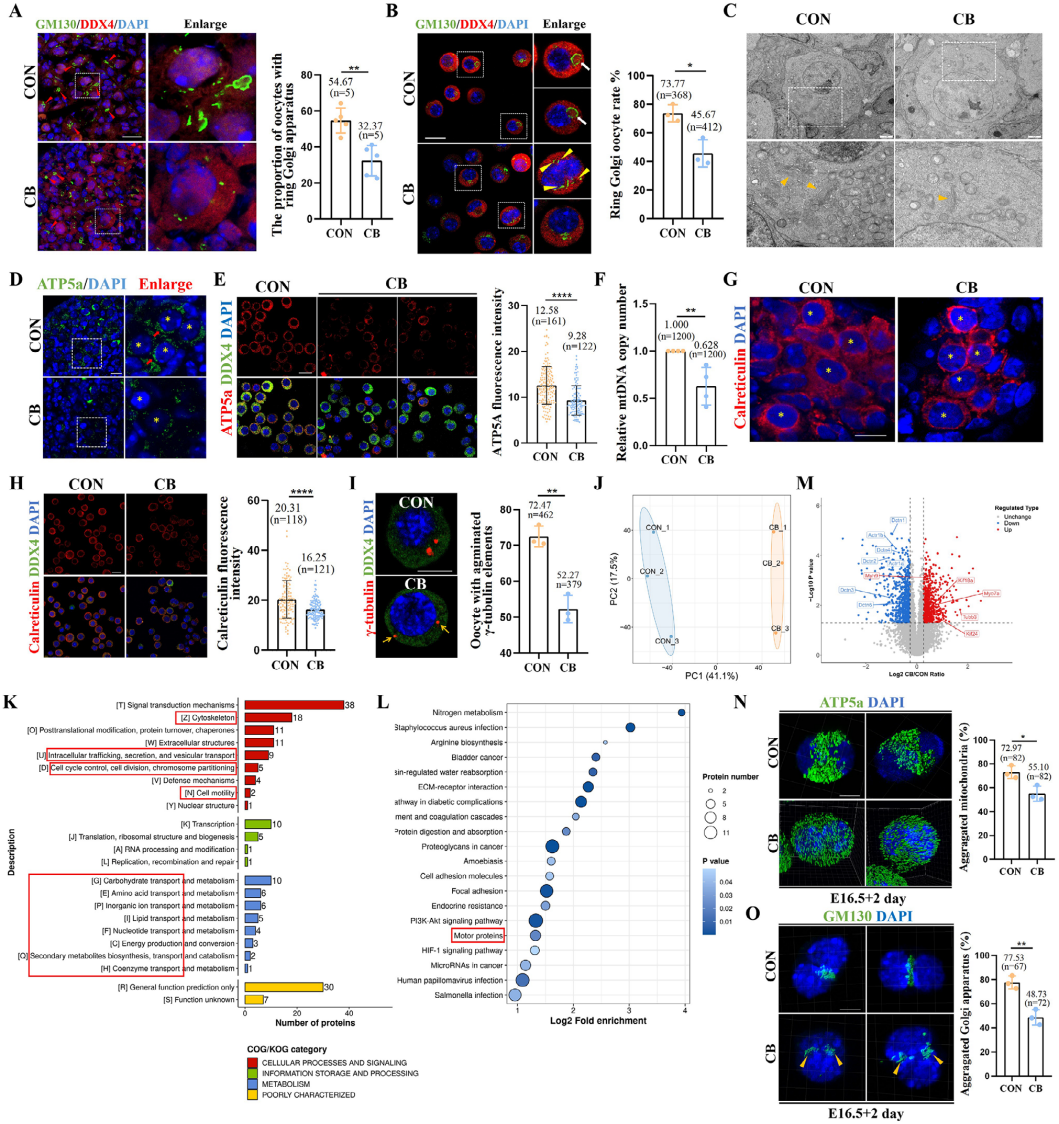
Microfilament depolymerization decreased the organelle content of primary oocytes. A) The GM130 staining and the proportion of oocytes with a ring Golgi apparatus in CON and CB treatment ovarian tissue slices, scale bar = 20 µm. B) The GM130 staining and the proportion of oocyte with a ring Golgi apparatus in oocytes isolated from CON and CB treatment group ovaries, scale bar = 20 µm. C) Transmission electron micrograph of oocyte in CON and CB treatment ovarian tissue slices, scale bar = 2 µm. D) The ATP5a staining in CON and CB treatment ovarian tissue slices, scale bar = 20 µm. E) The ATP5a staining and relative ATP5a fluorescence intensity in oocytes isolated from CON and CB treatment group ovaries, scale bar = 10 µm. F) Mitochondrial DNA (mtDNA) copy number in oocytes isolated from CON and CB treatment group ovaries. G) The calreticulin staining in CON and CB treatment ovarian tissue slices, scale bar = 20 µm. H) Relative calreticulin fluorescence intensity in oocytes from CON and CB ovarian tissue slices. I) The calreticulin staining and relative calreticulin fluorescence intensity in oocytes isolated from CON and CB treatment group ovaries, scale bar = 10 µm. J) Principal Component Analysis (PCA) results. K) COG analysis of differentially expressed proteins. L) KEGG analysis of differentially expressed proteins. M) A volcano plot demonstrates differential protein expression in CON versus CB group. N) The mitochondria localization and the proportion of multinucleated cyst with agminated mitochondria in multinucleated cyst of CON and CB treatment group after 2 days culture, scale bar = 10 µm. O) The Golgi apparatus localization and the proportion of multinucleated cyst with agminated Golgi apparatus in multinucleated cyst of CON and CB treatment group after 2 days culture, scale bar = 10 µm. ^*^ meant *p* < 0.05, ^**^ meant *p* < 0.01, and ^****^ meant *p* < 0.0001.

To further identify the role of microfilaments in oocyte differentiation, the E16.5 fetal mouse ovaries were treated with or without 2.5 µg mL^−1^ CB for 4 days. First, we detected the proportion of oocytes with a typical ring Golgi apparatus, which is a marker of the B‐body, in ovarian tissue slices. The CB treatment group showed a significantly lower proportion of oocytes that formed the typical ring Golgi apparatus (**Figure** **4**A). To confirm this finding, oocytes were isolated from enzymatically digested ovaries and stained with GM130. Consistent with the initial findings, the CB treatment group showed a lower number of ring Golgi apparatuses. Notably, in the multinucleated oocytes of the CB treatment group, the Golgi apparatus was scattered and appeared in multiple directions in the nucleus (Figure 4B). Transmission electron microscopy showed similar results (Figure 4C). These findings indicate that the destruction of microfilaments could inhibit the migration and fusion of the Golgi apparatus during oocyte differentiation. Next, we determined the mitochondrial content of differentiated oocytes in the two groups. Both tissue sections and isolated oocytes from the CB group displayed decreased mitochondrial content (Figure 4D,E). When the mitochondrial DNA copy number was determined to quantify the mitochondrial content in oocytes, oocytes in the CB group showed a lower mitochondrial DNA copy number, confirming this decrease (Figure 4F). Similarly, the ER content of oocytes in the CB treatment group was significantly reduced (Figure 4G,H). In differentiated oocytes, the centrosomes agminate and migrate to the Golgi ring apparatus.^[^ ^1a^ ^]^ However, the centriole distribution was more dispersed in the CB group than in the CON group (Figure 4I).

Proteomic profiling of the ovaries after 2 days of culture revealed distinct protein expression patterns between the CB‐treated and CON groups (Figure 4J). Gene ontology (GO) analysis showed that the differentially expressed proteins were mainly enriched in the cytoskeleton, intracellular trafficking, secretion, vesicular transport, cell division, cell motility, and a series of material transport (Figure 4K). Kyoto Encyclopedia of Genes and Genomes (KEGG) pathway analysis identified significantly altered motor protein‐related pathways (Figure 4L), and the volcano plot showed the motor proteins that had undergone expression changes (Figure 4M). Functional assays confirmed that organelle migration (mitochondria and Golgi) was markedly attenuated in the CB‐treated oocytes (Figure 4N,O). Furthermore, the expression of Myh9 (myosin IIA) and Myo7a (myosin VIIA), which underwent expression changes in the volcano map, was detected. Consistent with the sequencing results, the protein expression levels of myosin IIA and myosin VIIA increased after CB treatment, indicating the accuracy of the sequencing results (Figure S3A,B, Supporting Information). More importantly, we observed that, depolymerization of microfilaments was followed by a marked decrease in the protein expression level of the phosphorylated myosin II light chain, indicating a reduction in myosin II activity (Figure S3C, Supporting Information).

To further confirm these findings related to the role of microfilaments in oocyte differentiation, we utilized Jasplakinolide (Jas), an actin‐stabilizing drug. E16.5 fetal mouse ovaries were treated with or without 5 µM Jas for 4 days. Jas treatment altered the size of the ovaries and the morphology of oocytes (Figure S4A–C, Supporting Information), and F‐actin showed abnormal aggregation in both multinucleated cysts and oocytes (Figure S4D, Supporting Information). The polarity and volume of the oocytes showed significant changes (Figure S4E–G, Supporting Information). The proportion of double‐nucleated oocytes increased significantly (Figure S4H, Supporting Information). Subsequently, mitochondrial and ER contents were reduced in the Jas group (Figure S4I–M, Supporting Information). Golgi ring formation was also disrupted (Figure S4N,O, Supporting Information). As shown in Movies S5 and S7 (Supporting Information), live imaging of expelled multinucleated cysts revealed accelerated expulsion dynamics following Jas treatment. These results verify that microfilament dynamics are essential for organelle transport and positioning during oocyte differentiation.

### 2.5 ARP2/3 Complex Is Involved in Oocyte Differentiation

**Figure 5:**
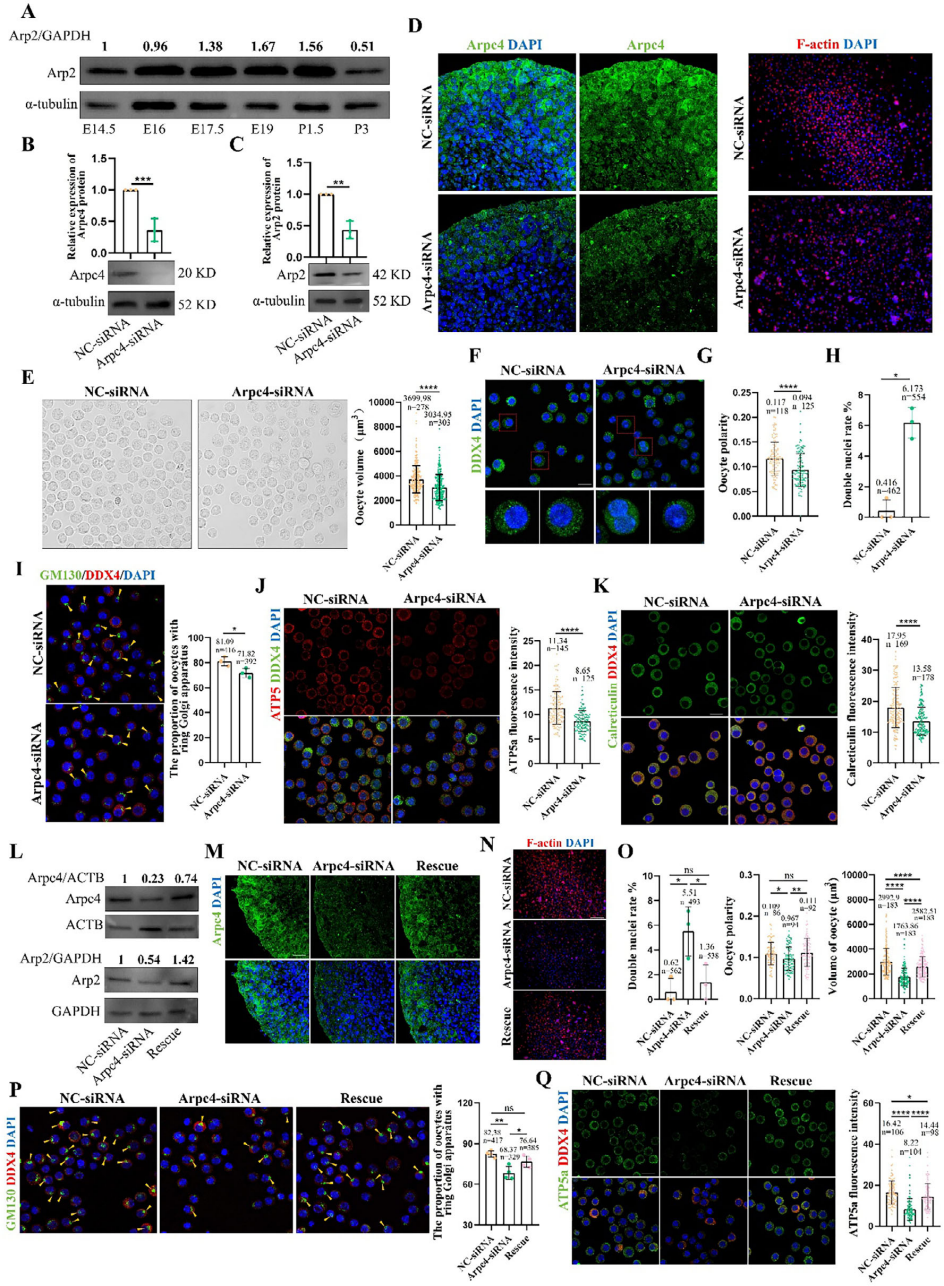
Arp2/3 complex participated in the oocyte differentiation process. A) The Arp2 protein expression levels during oocyte differentiation process. B,C) Arpc4 and Arp2 knockdown were achieved by using Arpc4 siRNA (*n* = 24 in each group). D) Arpc4 and F‐actin fluorescence intensity significantly decreased by using Arpc4 siRNA. E) Oocyte morphology and volume in NC‐siRNA and Arpc4‐siRNA group, scale bar = 50 µm. F) The DDX4 staining of oocytes in NC‐siRNA and Arpc4‐siRNA group, scale bar = 20 µm. G) The polarity degree of oocytes in NC‐siRNA and Arpc4‐siRNA group. H) The rate of oocytes with double nuclei in NC‐siRNA and Arpc4‐siRNA group. I) The proportion of oocyte with a ring Golgi apparatus in NC‐siRNA and Arpc4‐siRNA group, scale bar = 20 µm. J) The relative ATP5a fluorescence intensity in oocytes of NC‐siRNA and Arpc4‐siRNA group, scale bar = 20 µm. K) The relative calreticulin fluorescence intensity in oocytes of NC‐siRNA and Arpc4‐siRNA group, scale bar = 10 µm. L) The Arpc4 and Arp2 protein expression levels in NC‐siRNA, Arpc4‐siRNA and Rescue group. M,N) Arpc4 and F‐actin fluorescence intensity in NC‐siRNA, Arpc4‐siRNA and Rescue group. O) The rate of double nuclei, polarity degree and volume of oocytes in NC‐siRNA, Arpc4‐siRNA, and the Rescue group. P) The proportion of oocyte with a ring Golgi apparatus in NC‐siRNA, Arpc4‐siRNA, and the Rescue group, scale bar = 20 µm. Q) The relative ATP5a fluorescence intensity in oocytes of NC‐siRNA, Arpc4‐siRNA, and the Rescue group, scale bar = 20 µm. ^*^ meant *p* < 0.05, ^**^ meant *p* < 0.01, ^***^ meant *p* < 0.001, and ^****^ meant *p* < 0.0001.

The ARP2/3 complex plays a crucial role in mediating actin polymerization.^[^ ^19^ ^]^ The ARP2/3 protein was highly expressed during oocyte differentiation in ovaries (**Figure** **5**A). We disrupted the function of the ARP2/3 complex using a small interfering RNA (siRNA) targeting ARPC4 (a structural subunit essential for complex stability). The ARPC4 siRNA was injected into the ovaries of E15.5 fetal mice, and the ovaries were cultured for 5 days. ARPC4 and ARP2 were successfully knocked down 48 h after injection (Figure 5B,C), and the fluorescence intensity of ARPC4 and F‐actin was significantly decreased (Figure 5D). Knockdown of ARPC4 significantly impaired oocyte differentiation, manifesting as a smaller volume (Figure 5E), loss of polarity (Figure 5F,G), increased number of double‐nuclei oocytes (Figure 5H), and reduced the ring Golgi apparatus, mitochondria, and ER (Figure 5I–K). These defects were partially rescued by supplementing ARPC4 mRNA with ARPC4‐siRNA, which restored ARPC4 and ARP2 protein levels, F‐actin fluorescence intensity (Figure 5L–N), andthe differentiation quality of the primary oocytes (Figure 5O–Q). Similarly, after treatment with CK666, an inhibitor of the ARP2/3 complex, for 4 days, E15.5 fetal mice showed significantly reduced F‐actin polymerization (Figure S5A,B, Supporting Information), altered size of ovaries (Figure S5C,D, Supporting Information), disrupted oocyte volume and polarity, double‐nuclei oocytes (Figure S5E–G, Supporting Information), and organelle distribution and content defects (Figure S5H–K, Supporting Information). These findings emphasize the importance of microfilament dynamics in oocyte differentiation.

### 2.6 Microfilament Myosin II Regulates Oocyte Differentiation

Myosin II collaborates with microfilaments and plays a critical role in driving intracellular processes such as cytoplasmic flow and organelle movement. Proteome sequencing revealed that microfilament depolymerization altered the expression of myosin II family members and reduced myosin II activity (Figure S3, Supporting Information). On the basis of these results, we explored whether myosin II cooperates with microfilaments to regulate oocyte differentiation. The protein and mRNA expression levels of myosin II family members (myosin IIA, myosin IIB, and myosin IIC) were detected, and these proteins were found to be expressed during oocyte differentiation, with increased expression between E16.5 and P1.5 (Figure S6A–D, Supporting Information). Subcellular localization analysis of myosin II in multinucleated cysts demonstrated that myosin IIA co‐localized with microfilaments in the cortex and cytoplasm, myosin IIB was associated with the Golgi apparatus, and myosin IIC was distributed diffusely in the cytoplasm (Figure S6E–J, Supporting Information). A 2‐day treatment of E16.5 mouse ovaries with 50 µM blebbistatin (a total‐myosin II inhibitor) significantly reduced the phosphorylation level of the myosin II regulatory light chain, confirming effective inhibition of myosin II activity (Figure S7A, Supporting Information). After four days of blebbistatin treatment, the differentiated oocytes showed significant volume reduction (Figure S7B, Supporting Information), increased double‐nucleus rate, disrupted polarity (Figure S7C, Supporting Information), decreased ring Golgi apparatus formation (Figure S7D, Supporting Information), reduced ER and mitochondrial content (Figure S7E–G, Supporting Information), and significantly downregulated mRNA expression of oocyte development‐related genes (Figure S7H, Supporting Information). These findings suggest that inhibition of myosin II activity compromises the quality of oocyte differentiation.

**Figure 6:**
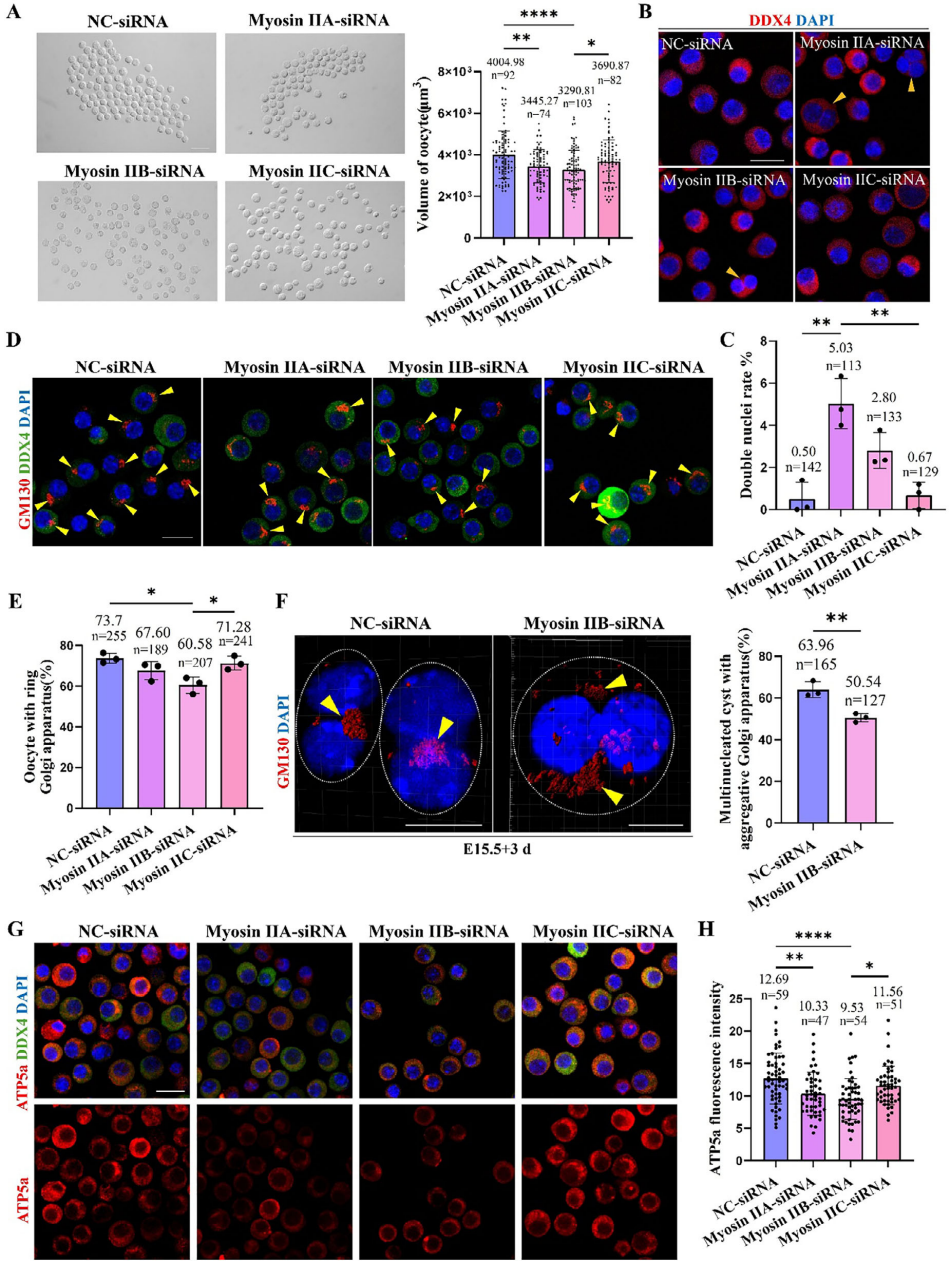
The myosin II family mediated microfilaments to regulate oocyte differentiation. A) Oocyte morphology and volume in four groups, scale bar = 100 µm. B,C) The staining of DDX4 and proportion of oocyte with double nuclei in four groups, scale bar = 20 µm. D,E) The staining of GM130 and proportion of oocyte with a ring Golgi apparatus in four groups, scale bar = 20 µm. F) The Golgi apparatus localization and the proportion of multinucleated cyst with agminated Golgi apparatus in multinucleated cyst of two groups, scale bar = 10 µm. G,H) The staining and relative ATP5a fluorescence intensity in oocytes of four groups, scale bar = 20 µm. ^*^ meant *p* < 0.05, ^**^ meant *p* < 0.01, and ^****^ meant *p* < 0.0001.

Considering the different localization of the three myosin II isoforms in multinucleated cysts, different subtypes may have different regulatory roles during oocyte differentiation. Therefore, siRNA was used to specifically knock down the three myosin II isoforms in the ovaries. After microinjection, the siRNA was uniformly distributed throughout the ovary (Figure S8A, Supporting Information), and the three myosin II subtypes were specifically knocked down after two days (Figure S8B–G, Supporting Information). The results showed that knockdown of myosin IIA and myosin IIB significantly reduced oocyte volume (**Figure** **6**A). Myosin IIA knockdown led to the emergence of more double‐nucleated oocytes (Figure 6B,C). Myosin IIB knockdown notably hindered ring Golgi apparatus formation in oocytes and impaired Golgi migration and fusion in multinucleated cysts (Figure 6D–F). Additionally, myosin IIA and IIB knockdown decreased oocyte mitochondrial content (Figure 6G,H). These results further indicated that myosin II family members work with microfilaments to regulate oocyte differentiation, with each subtype having distinct effects.

Next, we further investigated the mechanism through which microfilament depolymerization impacted the activity of myosin II. Myosin II activity is predominantly regulated by the RhoA‐ROCK signaling pathway, which is known to be highly responsive to intracellular mechanical alterations.^[^ ^36^ ^]^ CB treatment two days resulted in a significant upregulation of p‐RhoA (the inactive form of RhoA), as well as a significant downregulation in the expression levels of p‐ROCK1, p‐ROCK2, and p‐MYL in ovaries (Figure S9A,B, Supporting Information). The down‐regulation of p‐ROCK1, p‐ROCK2, and p‐MYL protein expressions by CB treatment were significantly reversed by co‐treatment with 2 mm Pentanoic acid (ROCK activator) (Figure S9C,D, Supporting Information). Meanwhile, compared with the CB treatment group, the addition of Pentanoic acid partially increased the content of mitochondria and ring Golgi apparatus in oocytes (Figure S9E–H), and the volume, double nuclei rate and polarity of oocytes had also partially recovered (Figure S9I–K, Supporting Information). Further, the addition of Pentanoic acid significantly increased the content of F‐actin in the ovaries compared with the CB treatment group (Figure S9L, Supporting Information). These results indicate that microfilament depolymerization regulates myosin II activity through RhoA‐ROCK signaling and further affects the quality of oocyte differentiation.

### 2.7 Depolymerization of Microfilaments Impairs PF Formation and Subsequent Follicular Development

**Figure 7:**
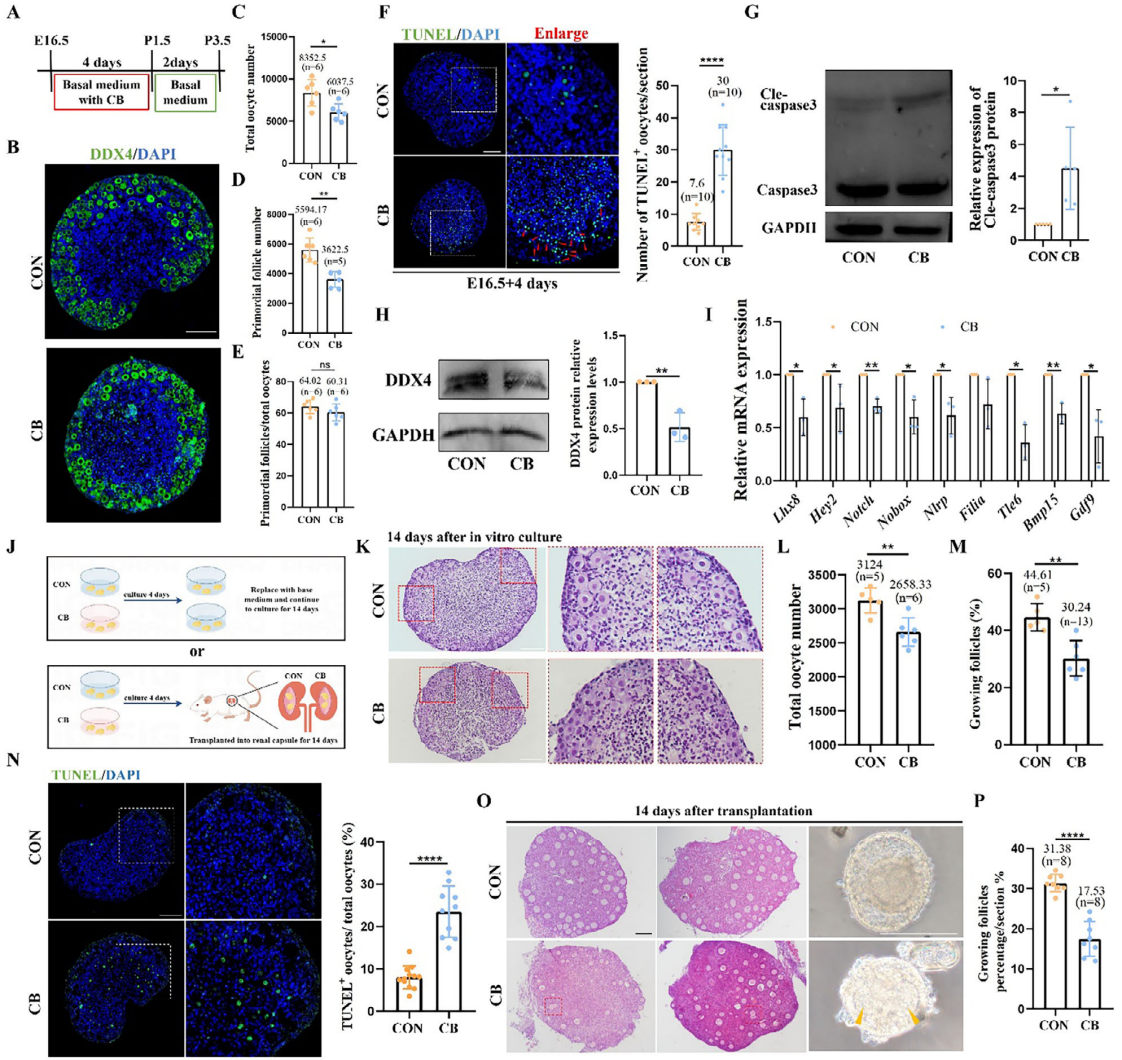
Depolymerization of microfilaments decreased the formation of primordial follicles and hindered follicular development. A) The schematic diagram of ovaries treatment schedules. B) The DDX4 staining in CON and CB treatment ovarian tissue slices, scale bar = 100 µm. C) The number of total oocytes in CON and CB treatment ovaries. D) The number of primordial follicles in CON and CB treatment ovaries. E) The proportion of primordial follicles in total oocytes. F) The number of TUNEL‐positive oocytes in CON and CB treatment ovarian tissue slices, scale bar = 50 µm. G) The Cle‐caspase3 protein expression levels in CON and CB treatment group (*n* = 30 in each group). H) The DDX4 protein expression levels in CON and CB treatment group (*n* = 18 in each group). I) qRT‐PCR analysis of oocyte‐specific genes mRNA expression in CON and CB treatment ovaries, *Ddx4* was used as internal reference gene (*n* = 18 in each group). J) The schematic diagram of ovaries subsequent treatment schedules. K) The status of oocyte development in CON and CB treatment ovaries after 14 days in vitro culture, scale bar = 100 µm. L) Total oocytes in CON and CB treatment group ovaries. M) The proportion of growing follicles in CON and CB treatment group ovaries. N) The proportion of TUNEL positive oocytes in CON and CB treatment ovarian tissue slices, scale bar = 100 µm. O) The status of oocyte development and follicle morphology in CON and CB treatment ovaries after 14 days in vivo transplantation, scale bar = 100 µm. P) The proportion of growing follicles in CON and CB treatment ovaries after 14 days in vivo transplantation. ^*^ meant *p* < 0.05, ^**^ meant *p* < 0.01, ^***^ meant *p* < 0.001, ns meant *p* > 0.05.

Our findings demonstrated the importance of microfilaments in oocyte differentiation. We further explored microfilament depolymerization in PF assembly and subsequent development. The ovarian treatment process is shown in **Figure** **7**A. Quantitative analysis revealed a significant decrease in both the total oocyte number and PF count in the CB‐treated group, but no significant difference was observed in the PF count:total oocyte ratio (Figure 7B–E). Next, we found that the number of apoptotic germ cells and Cle‐caspase3 protein expression levels significantly increased in the CB treatment group (Figure 7F,G). Meanwhile, DDX4 protein was significantly downregulated after CB treatment (Figure 7F), and key oocyte developmental genes exhibited reduced transcriptional activity (Figure 7I). Considering the essential role of pre‐granulosa cells in follicle formation, we detected their proliferation and differentiation after 4 days of CB treatment. Previous studies have shown that FOXL2^+^ pre‐granulosa cells surround oocytes and form PFs.^[^ ^20^ ^]^ In our study, the number of FOXL2^+^ pre‐granulosa cells decreased slightly, but the difference was not statistically significant (Figure S9A–C, Supporting Information). Similarly, Ki67 and proliferating cell nuclear antigen (PCNA) staining showed no significant changes in cell proliferation in whole ovaries (Figure S9D–I, Supporting Information). These results indicate that the reduction in PFs during CB treatment was mainly caused by apoptosis of germ cells.

To explore whether impaired oocyte differentiation affects subsequent follicular development, we subjected the ovaries from the CON and CB treatment groups to extension in vitro for 14 days or transplanted them under the renal capsule for 14 days (Figure 7J). After 14 days of in vitro culture, the CON group showed a higher quantity of PFs that had been activated and developed as well as a greater growth status of the oocytes (Figure 7K–M). In contrast, the ovaries of the CB group displayed increased oocyte apoptosis, which may be linked to lower oocyte quality (Figure 7N). Renal subcapsular transplantation of ovaries from the CON and CB groups was performed for 14 days. The results were similar to those obtained in vitro; the CB group showed fewer developing follicles and multiple‐oocyte follicles (Figure 7O,P). These results indicate that microfilaments play an important role in oocyte differentiation, and a reduction in oocyte differentiation quality further affects subsequent follicle activation and development.

### 2.8 Interference with Oocyte Differentiation Impairs Oocyte Maturation Quality

**Figure 8:**
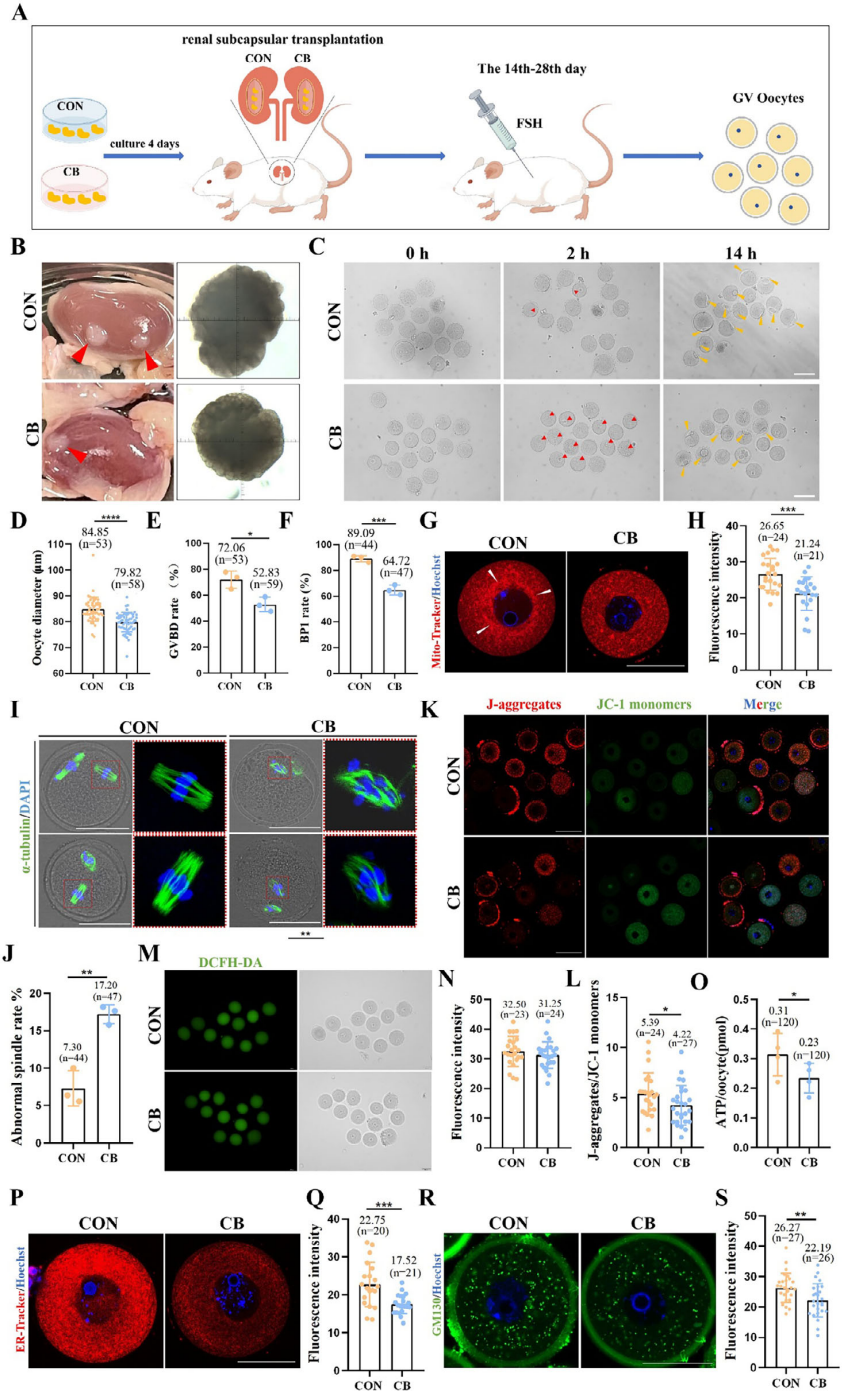
Interference with oocyte differentiation impaired the quality of oocyte maturation. A) The schematic diagram of treatment for CON and CB group ovaries. B) The ovarian morphology in CON and CB groups after 28 days of renal subcapsular transplantation. C) The oocyte meiosis process after 2 and 14 h in vitro culture in CON and CB treatment groups, scale bar = 100 µm. D) The diamater of GV oocytes in CON and CB groups after 28 days of renal subcapsular transplantation. E) The GVBD rate of oocytes in CON and CB groups. F) The PB1 rate of oocytes in CON and CB groups. G) The typical images of mitochondrial distribution (Mito‐Tracker staining) of oocyte in CON and CB groups, scale bar = 50 µm. H) Quantitative analysis of relative red fluorescence intensity in G. I) The typical images of MII spindle in CON and CB groups, scale bar = 50 µm. J) The abnormal spindle rate of oocytes in CON and CB groups. K) The typical images of mitochondrial membrane potential (JC‐1 staining) in oocytes in CON and CB groups, scale bar = 100 µm. L) Quantitative analysis of mitochondrial membrane potential in K. M) The typical images of ROS (DCFH‐DA staining) in oocytes in CON and CB groups, scale bar = 50 µm. N) Quantitative analysis of relative fluorescence intensity in M. O) The ATP levels of oocytes in CON and CB groups. P) The typical images of ER distribution (ER‐Tracker staining) in oocytes in CON and CB groups, scale bar = 50 µm. Q) Quantitative analysis of relative fluorescence intensity in P. R) The typical images of Golgi apparatus distribution (GM130 staining) in oocytes in CON and CB groups, scale bar = 50 µm. S) Quantitative analysis of relative fluorescence intensity in R. ^*^ meant *p* < 0.05, ^**^ meant *p* < 0.01, ^***^ meant *p* < 0.001, ns meant *p* > 0.05.

Our study demonstrated that interference with oocyte differentiation impeded the activation of PFs and their subsequent development. To explore the long‐term influence of early differentiation on oocyte maturation quality, we collected oocytes from CON and CB‐treated ovaries 28 days after renal subcapsular transplantation (**Figure** **8**A–C). The oocytes in the CB treatment group had a significantly smaller diameter than those in the CON group (Figure 8D). The germinal vesicle breakdown and first polar body (PB1) excretion rates were also markedly reduced in the CB‐treated group (Figure 8C,E,F), indicating poor oocyte quality. Furthermore, we analyzed mitochondrial activity and content‐related indicators in the GV‐stage oocytes. Mitochondrial proliferation is accomplished by the division of existing mitochondria; thus, the number of mitochondria is determined on the basis of the initial mitochondrial content. In contrast, mitochondrial membrane potential (MMP), mitochondrial distribution, and function are critical indicators of oocyte maturation.^[^ ^21^ ^]^ In the CON group, mitochondria clustered around the nucleus, with higher mitochondrial content, MMP, and adenosine triphosphate (ATP) levels than in the CB‐treated group (Figure 8G,H,K,L,O). However, ROS levels showed no significant differences between the two groups (Figure 8M,N). Furthermore, the proportion of MII abnormal spindles significantly increased in the CB‐treated group (Figure 8I,J). The oocytes of the CB treatment group showed fewer ER and Golgi apparatuses (Figure 8P–S). These results suggested that interference with early oocyte differentiation impairs oocyte maturation quality.

## 3 Discussion

**Figure 9:**
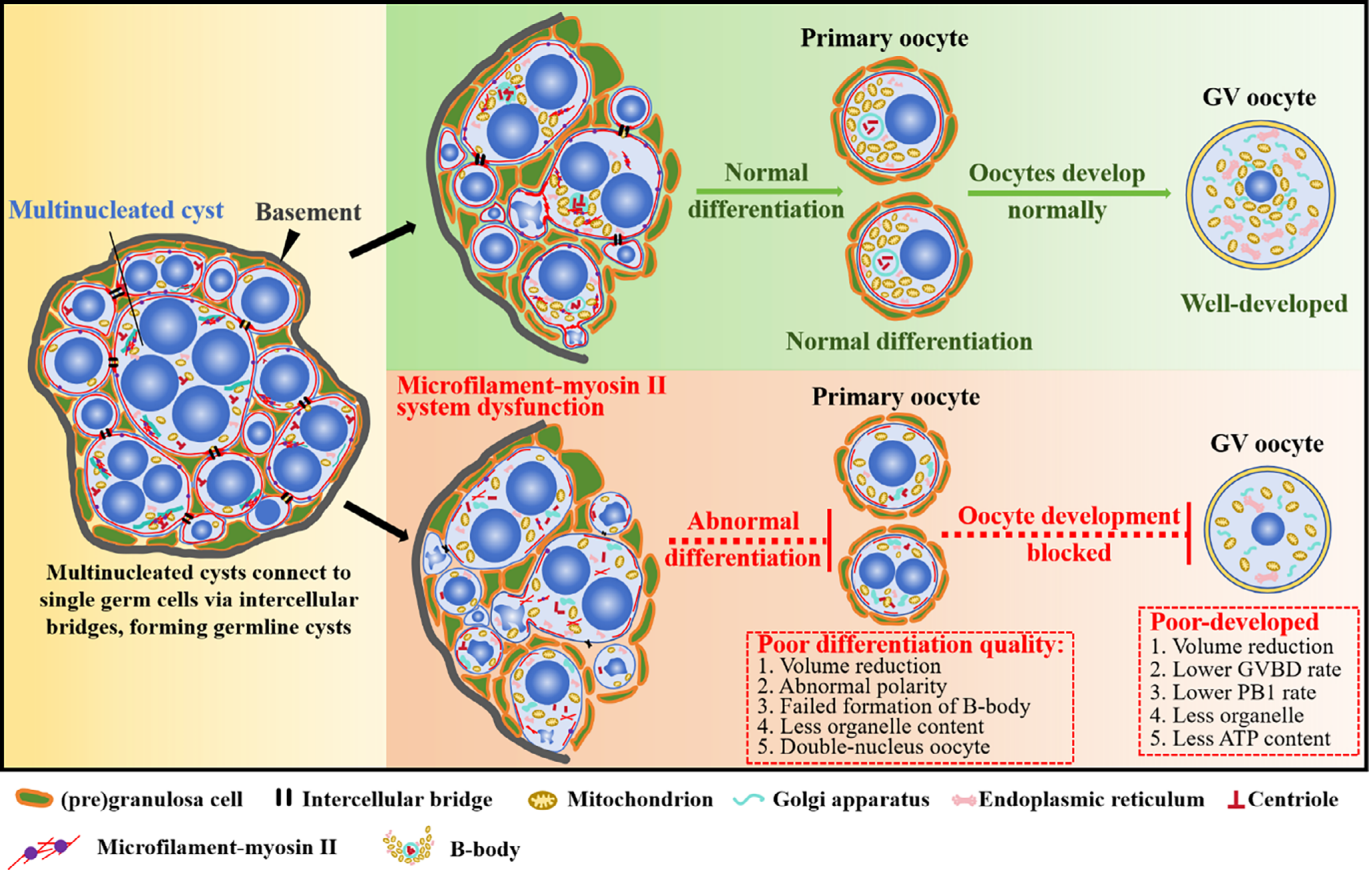
Microfilament‐myosin II regulates the differentiation of multinucleated cysts into oocytes and influences oocytes developmental potential in mouse.

In this study, we provided new evidence identifying multinucleated cysts as precursors for oocyte differentiation. Within these cysts, organelles migrate and aggregate to form B‐bodies and establish early cellular polarity. Concurrently, excess nuclei are expelled, enabling cyst‐to‐oocyte differentiation. The microfilament myosin II plays a crucial role in these processes. Any disruption of these dynamic changes or activities affects the quality of oocyte differentiation. Furthermore, poor oocyte differentiation negatively hinders the subsequent development and maturation of oocytes (**Figure** **9**).

The number of PFs in the PF pool determines the reproductive lifespan of female mammals. In female fetal ovaries, only a small proportion of PGCs eventually differentiate into primary oocytes and assemble with the surrounding pre‐granulosa cells, establishing PFs. The pool of dormant PFs functions as an ovarian reserve that is essential for maintaining ovarian function and supporting egg production.^[^ ^1^, ^22^ ^]^ In *Drosophila*, mice, and humans, only a minor proportion—≈15–20%—of germ cells eventually develop into primary oocytes, while the other germ cells undergo programmed cell death.^[^ ^2^, ^23^ ^]^ However, the reasons that decide the different differentiation fates of germ cells during mammalian oocyte differentiation remain to be elucidated.

Previous studies have identified branched germline cyst structures during gametogenesis in several species.^[^ ^24^ ^]^ In the fetal female germline, 16.8% of the germ cells connect with other germ cells through three or four intercellular bridges and are named branching germ cells. These branching germ cells, with the most intercellular bridges, are preferentially protected from cell death and accumulate the cytoplasm and organelles from sister germ cells to become primary oocytes.^[^ ^6^ ^]^ In the present study, we observed that germ cells in fetal mice exist in two forms: single germ cells and multinucleated cysts. The multinucleated cysts are connected to single germ cells by intercellular bridges. Furthermore, we found that branching germ cells with 3–4 intercellular bridges were often multinucleated cysts rather than single germ cells. Coincidentally, when oogonia had just finished mitosis and oocyte differentiation had not yet begun, the percentage of multinucleated cysts in total germ cells reached 18.23% by E14.5, and gradually decreased during subsequent development. This proportion appeared to correspond to the proportion of germ cells that eventually differentiate into oocytes. Similarly, multinucleated cysts have been found during the formation of PFs in the human fetal ovary.^[^ ^1d^ ^]^ However, the previous study reported that not all multinucleated cysts were connected to other germ cells by intercellular bridges. A possible explanation for this might be that previous studies often used ovarian tissue section images, leading to incomplete observations. In our study, the digestion of ovaries into a single‐cell suspension solved this problem and facilitated a more comprehensive analysis.

Intercellular bridges are structures conserved during gametogenesis in several species.^[^ ^8^ ^]^ In mammals, the bridge is composed of general cell division components and additional germ cell‐specific factors such as TEX14 and ARF6.^[^ ^9^, ^25^ ^]^ They connect the cytoplasm of neighboring germ cells and can be 0.5–3 µm in diameter, allowing various forms of molecular and mitochondrial transit.^[^ ^1^, ^2^ ^]^ Germ cells are currently thought to obtain cytoplasmic components from other sister germ cells through intercellular bridges, facilitating their eventual differentiation into oocytes.^[^ ^26^ ^]^ This would imply that these bridges play a crucial and even decisive role in the cytoplasmic transport process, and any disruption of the bridge would result in a breakdown in the communication between germ cells. However, TEX14 knockout in female mice did not result in infertility, suggesting that intercellular bridges may not play a decisive role in germ cell connectivity and oocyte differentiation or in oocyte quality and functionality, and the transport of cytoplasmic components during oocyte differentiation may also be mediated by other mechanisms.^[^ ^10a,b^ ^]^ In the present study, we found that the multinucleated cyst itself has a large number of cytoplasmic components, and its own cytoplasm may protect the multinucleated cyst from being unable to complete the cytoplasm reserve and differentiation due to the breakdown of the intercellular bridge. The organelles inside multinucleated cysts underwent notable spatial changes from E15.5‐P1; the Golgi apparatus, centrioles, and mitochondria gradually migrated from the perinuclear location to the middle of the two nuclei and fused together. This migration and fusion formed a complex structure that is very similar to the B‐body or its precursors in primary oocytes. More importantly, the volume of the Golgi apparatus in early stage (E15.5) multinucleated cysts and P1 primary oocytes was almost the same, which may imply that the Golgi apparatus in oocytes does not need to be acquired from other single germ cells through the intercellular bridge, and is entirely supplied by the multinucleated cyst, which is similar to the results obtained in humans.^[^ ^1d^ ^]^ However, the Golgi apparatus often presents as a long strip or ring, which may not be conducive for passing the narrow intercellular bridge to transit. The presence of multinucleated cysts may explain the absence of infertility in offspring when the intercellular bridge is broken. Research has shown that that the expressions of nurse cell and pro‐oocyte genes diverge after nurse cells initiate cytoplasmic transfer, and although the nurse cells could also enter meiosis, they were much weaker than the pro‐oocytes.^[^ ^2^ ^]^ Similarly, in our findings, the process of nuclear meiosis in multinucleated cysts usually proceeded more rapidly than that in single germ cells, and multinucleated cysts often exhibited synchronized nuclei, as in humans.^[^ ^1d^ ^]^

The actin cytoskeleton is the primary force‐generating machinery in the cell. It maintains the cell shape and mechanical properties of the cell surface, drives intracellular motility, and is involved in a crucial step in cell division.^[^ ^27^ ^]^ The process of differentiation from multinucleated cyst to primary oocytes is similar to that of spermatogenesis. Secondary spermatocytes produce two connected haploid spermatids, which further differentiate under the action of actin filaments and actin‐based molecular motors, shedding undesired cytoplasm in the form of a residual body and inheriting mitochondria, Golgi‐derived organelles, and the nucleus.^[^ ^28^ ^]^ Similarly, multinucleated cysts shed undesired residues in the presence of microfilaments, and these residues often exhibit intense lysosomal staining, indicating that they were degraded by lysosomes, similar to the acidification of nurse cells.^[^ ^2^, ^29^ ^]^ On the other hand, microfilaments can drive intracellular cytoplasm flow and affect organelle localization.^[^ ^30^ ^]^ The mechanism underlying cytoplasmic flow is closely associated with microfilaments and their interactions with the motor protein myosin. Actin filaments function not only as tracks for myosin to transport cellular cargo, but also as force generators through their interaction with myosin, producing the driving force required for cytoplasmic streaming and establishing a dynamic intracellular environment.^[^ ^18^, ^31^ ^]^ In our study, depolymerization and over‐polymerization of microfilaments both disturbed B‐body establishment, polarity, and organelle content in primary oocytes. Proteomic sequencing of ovaries treated with CB showed that intracellular material transport and cell motility‐related biological events were significantly affected, and the findings also indicated differential protein enrichment into motor protein‐related pathways. Motor proteins mediate the transport of intracellular material and organelles.^[^ ^32^ ^]^ For example, during *Drosophila* oogenesis, myosin II activity is required for rapid cytoplasmic transport. Oocytes with lower myosin II activity fail to reach full size due to the “dumping” defect (a rapid stage of cytoplasmic transport from the nurse cell).^[^ ^17^ ^]^ However, different subtypes of myosin II have different functions. Knockdown of myosin IIA led to a higher incidence of binuclear oocytes (incompletely differentiated multinuclear cyst) suggesting that myosin IIA knockdown may hinder the extrusion process of multinuclear syncytia, thereby impeding complete differentiation. In contrast, knockdown of myosin IIB disrupted the formation of the ring‐shaped Golgi apparatus, likely due to impaired migration and fusion of Golgi organelles within the cyst. This is consistent with the observed co‐localization of myosin IIB with the Golgi apparatus, indicating that Golgi migration in multinuclear syncytia depends on myosin IIB‐mediated transport. Further experiments confirmed that myosin IIB knockdown indeed reduced the migration rate of Golgi organelles within the cyst. These results demonstrate that different myosin II isoforms play distinct roles in the differentiation of multinuclear cyst into oocytes. Existing literature indicates that most cells express more than one type of myosin II, and that the localization of myosin II varies across cell types. Their distinct subcellular distributions, expression levels, and dynamic properties may account for their diverse biological functions.^[^ ^37^ ^]^ For example, during the exocytotic process of membrane repair, myosin IIB was primarily at the subplasmalemmal cortex and myosin IIA was concentrated at the trans‐Golgi network.Myosin IIB was required for exocytosis and therefore cell membrane repair itself and that myosin IIA was required in facilitation of cell membrane repair at repeated wounds. These are consistent with their distinct roles in vesicle trafficking in cell membrane repair (14 617 807). Our research also demonstrated that myosin II activity is crucial for oocyte differentiation. We further found that destruction of the microfilament cytoskeleton led to the failure of motor protein transport and reduced activity, inhibiting the migration and fusion of organelles in multinucleated cysts. This resulted in failure to establish the B‐body in the primary oocyte. Microfilaments are the structural basis of intercellular bridges in *Drosophila*, and this phenomenon has also been observed in mice.^[^ ^15^, ^33^ ^]^ The degradation of microfilaments also caused the destruction of intercellular bridges, which hindered the transport of cytoplasm from single germ cells to multinucleated cysts, thereby further reducing the contents of mitochondria and ER in primary oocytes. The quality of oocyte differentiation determines subsequent oocyte development. B‐bodies are associated with RNA metabolism, and insufficient cytoplasmic components lead to subsequent oocyte dysplasia, which was verified in our study.^[^ ^1^, ^3^ ^]^ The interference of dynamic changes in the microfilaments results in more multinucleated oocytes. These conjoined and binucleated giant oocytes can potentially lead to chromosomal disorders in the embryos.^[^ ^34^ ^]^

## 4 Conclusions

In conclusion, we provide new evidence proving that multinucleated cysts act as precursors for oocyte differentiation. Furthermore, our study revealed that microfilament myosin II is crucial for establishing B‐body and polarity in primary oocytes, while also regulating the differentiation of multinucleated cysts into oocytes. In addition, our findings indicated that the differentiation quality of primary oocytes directly determines subsequent oocyte development and maturation quality.

## 5 Experimental Section

### Animals

ICR (12 weeks) were obtained from CHENGDU DOSSY EXPERIMENTAL ANIMALS CO., LTD., Chengdu, China. Mice were maintained under a 12‐hour light/dark cycle at a temperature of 21–25 °C with free access to food and water. Female mice were cohabitated with male mice overnight, and vaginal plugs were examined the following morning. The presence of a vaginal plug was designated as embryonic (E) 0.5 day. The day of parturition was recorded as postnatal (P) day 1. All experiments in this research were complied with the principles and guidelines for the use of laboratory animals and approved by the Institutional Animal Care and Use Committee of the College of Veterinary Medicine, Northwest A&F University (No.2021070712).

### Antibodies and Chemicals

DMEM/F‐12 (L340KJ), Insulin‐transferrin and selenium (S450J7), BSA (S475T7), and Penicillin‐streptomycin (S110JV) were purchased from BasalMedia Technologies (Shanghai, China). Cytochalasin B (HY‐16928), Jasplakinolide (HY‐P0027), CK666 (HY‐16926), Blebbistatin (HY‐13441) and pentanoic acid (HY‐N6056) were purchased from MCE (Beijing, China). Phalloidin‐FITC (P5282) was purchased from Sigma (MO, United States). Anti‐DDX4/MVH antibody (ab270534, ab27591), Anti‐FOXL2 antibody (ab246511), Anti‐Ki67 antibody (ab11580), Anti‐PCNA antibody (ab92552), Anti‐ATP5a antibody (ab176569), Anti‐GM130 antibody (ab52649), Anti‐gamma Tubulin antibody (ab179503), Anti‐LC3B antibody (ab192890), Anti‐p62 antibody (ab109012), Anti‐Calreticulin antibody (ab92516), Goat anti‐rabbit IgG (Alexa Fluor 488) (ab150077), Goat anti‐mouse IgG (Alexa Fluor 594) (ab150120) and Anti‐MYL9 (pS20) + MYL12A (pS19) + MYL12B (pS20) antibody (ab316750) were purchased from Abcam (MA, United States). MYH9 Polyclonal antibody (11128‐1‐AP), MYH10‐Specific Polyclonal antibody (19673‐1‐AP), MYH14 Polyclonal antibody (20716‐1‐AP), MYO7A Polyclonal antibody (20720‐1‐AP), TEX14 Polyclonal antibody (18351‐1‐AP), ARP2 Polyclonal antibody (10922‐1‐AP), ARPC4 Polyclonal antibody (10930‐1‐AP), ROCK1 Polyclonal antibody (21850‐1‐AP), ROCK2 Monoclonal antibody (66633‐1‐Ig) and RHOA Polyclonal antibody (10749‐1‐AP) were purchased from Proteintech (Wuhan, China). Phospho‐ROCK2 (Ser 1366) Antibody (TA7143) and Phospho‐ROCK1 (Thr455+Ser456) Rabbity pAb (PC4630) were purchased from Abmart (Shanghai, China). Phospho‐RhoA (Ser188) Antibody （AF8020） was purchased from Affinity Biosciences (Jiangsu, China). Donkey anti‐Rabbit IgG (A21206) and Donkey anti‐Mouse IgG (A32766) were purchased from Life Technologies (Beijing, China). One Step TUNEL Apoptosis Assay Kit (C1088), DAPI (C1002), Lyso‐Tracker Red (C1046), Mitochondrial membrane potential assay kit with JC‐1 (C2006), Golgi‐Tracker Red (C1043), Reactive Oxygen Species (ROS) Assay Kit (S0033S), Mito‐Tracker Red CMXRos (C1049B) and Enhanced ATP Assay Kit (S0027) were purchased from Beyotime (Shanghai, China). All other chemicals used in this study were purchased from Sigma unless otherwise stated.

### Ovary Isolation and Culture

Ovaries were separated from fetal mouse ovarian capsules by tweezers under a microscope and washed in Phosphate Buffered Saline (PBS). Then, 3–5 ovaries were transferred and cultured into 35 mm dishes with 1.2 mL ovarian culture medium at 37.5 °C, 5% CO_2_ and saturated humidity. ovarian culture medium included DMEM/F‐12, 0.3% BSA, 100X ITS and 100X Penicillin‐streptomycin. The final working concentration of Cytochalasin B (CB), Jasplakinolide (Jas), CK666, Blebbistatin and Pentanoic acid in ovarian culture medium were 2.5 µg mL^−1^, 5, 200, 50 µm, and 2 mm, respectively.

### Histological Sections and Follicle Counts

Ovaries were fixed in 10% Neutral formalin fixative at least 12 h, embedded in paraffin, and serially sectioned at 5 µm. The ovarian sections were stained with Hematoxylin‐Eosin (HE) Stain Kit (G1120, Solarbio) or Anti‐DDX4/MVH antibody, and the number of follicles per ovary was determined by counting in every fifth section. To estimate the total number of oocytes in each ovary, the sum was multiplied by five.

### Ovarian Single‐Cell Suspension Preparation

Ovaries were washed in BPS, then transferred into 1.5 mL tubes containing 0.125% trypsin (per ovary, 200 µL 0.125% trypsin) and incubated at 37.5 °C for 10–15 minutes. During incubation, the ovarian digestion was accelerated by repeatedly pipetting. Digestion was terminated by adding the same volume of 10% FBS‐PBS when the ovaries were significantly smaller. The supernatant was removed by centrifugation at 2500 rpm for 3 min. Following this, 500 µL of DMEM/F‐12 was added to resuspend the cell pellets, and centrifuged again under the same conditions. Finally, ovarian culture medium was added to resuspend the pellets and obtain single‐cell suspensions.

### Oocyte Polarity Analysis

Oocytes were isolated from the single‐cell suspensions under a microscope. The oocytes were stained with Anti‐DDX4/MVH antibody and DAPI to distinguish the nucleus and the cytoplasm. oocytes were then examined and imaged using a confocal microscope. Then, the distance between the nucleus midpoint and the oocyte midpoint was calculated. Degree of oocyte polarity = Distance between nuclear midpoint and oocyte midpoint/oocyte diameter.

### Mitochondrial DNA (mtDNA) Copy Number Measurement

For each biological replicate, 500 oocytes were isolated from single cell suspensions in the CON and CB groups under the microscope, respectively. QIAamp DNA Micro Handbook (56 304, QIAGEN) was used to extract oocyte total DNA. Next, a real‐time PCR reaction was performed to analyze the mtDNA copy number change. The primer sequences for the respective genes are listed in **Table** **2**.

### TUNEL Staining

The ovaries were subjected to fixation, embedding, and sectioning into 5 µm‐thick slices. According to the manufacturer's instructions for the one‐step TUNEL cell apoptosis detection kit (C1086, Beyotime, Shanghai, China), the sections were stained with TUNEL. The largest section of the ovary was selected for the analysis of cell apoptosis.

### Renal Capsule Transplantation

Ovarian transplantation under the renal capsule was performed following a previously established protocol.^[^ ^35^ ^]^ In brief, healthy 8‐week‐old female mice were anesthetized with 0.14 mL/10 g pentobarbital sodium. Following fur removal and surgical disinfection, the ovaries were surgically removed, and the kidneys were exposed. Then, several ovaries (3–5) from the CON and CB treatment groups were transplanted, respectively, under the left or right kidney capsules in the same recipient. Upon completion of the transplantation, the kidneys were repositioned into the abdominal cavity, and the incision was closed with sutures.

### Oocyte Collection and Culture

Renal capsule transplantation was conducted using ovaries from the CON and CB treatment groups for a duration of 28 days. Starting from the 14th day post‐transplantation, mice were administered daily intraperitoneal injections of 2 IU FSH for a consecutive period of 14 days. After 28 days of transplantation, the mice were euthanized via cervical dislocation, and the ovaries were carefully excised from the renal capsule. Germinal vesicle (GV)‐stage oocytes were collected from the ovarian puncture by a syringe needle. The GV oocytes were cultured in M2 medium for 2 or 14 h, corresponding to reaching the germinal vesicle breakdown (GVBD) stage or the metaphase II (MII) stage, respectively.

### Mito‐Tracker Red Staining, ER‐Tracker, and Lyso‐Tracker Red Staining

For Mito‐Tracker Red, ER‐Tracker, and Lyso‐Tracker Red staining, the live oocytes were incubated with Mito‐tracker Red (1:1000 dilution), ER‐tracker Red (1:1000 dilution), or Lyso‐tracker Red (1:1000 dilution) in M2 medium at 37 °C and 5% CO_2_ for 30 minutes. Then, the oocytes were washed three times with M2 medium, and finally, examined with a confocal microscope (LEICA TCS SP8).

### Mitochondrial Membrane Potential (MMP) and ROS Measurement

For MMP measurement, the living oocytes were incubated with JC‐1(1:200 dilution) in M2 medium for 30 minutes at 37 °C and 5% CO_2_. Finally, the oocytes were examined with a confocal microscope (LEICA TCS SP8). The MMP level was calculated as the ratio of red (J‐aggregate) fluorescence intensity to green (JC‐1 monomer) fluorescence intensity.

For ROS measurement, the living oocytes were incubated with or DCFH‐DA (1:1000 dilution) in M2 medium for 30 min at 37 °C and 5% CO_2_. Finally, the oocytes were examined with a confocal microscope (LEICA TCS SP8).

### ATP Content Measurement

The ATP content was measured according to the manufacturer's protocol. For each biological replicate, 60 µL of ATP‐releasing agent was added to lyse 30 oocytes. Hundred microliters of ATP working solution was dispensed into each well of a 96‐well culture plate, followed by the addition of 20 µL of sample or standard to the respective wells. A multimode microplate reader (Tecan Life Sciences) was used to detect fluorescent values. ATP levels in single oocytes were calculated based on the standard curve derived using internal standards.

### siRNA and mRNA Infection

The siRNA and mRNA used in experiment were purchased from GenePharma (Shanghai, China). The siRNAs are exhibited in **Table** **1** . In vivo‐jetPEI transfection reagent was purchased from Polyplus (101 000 040, Illkirch‐Graffenstaden, France). Following the manufacturer's protocols, diluting 16.5 µg of siRNA or mRNA into 10 µL of 10% glucose and adding sterile water to 20 µL, vortex gently and spin down. Then, diluting 2.64 µL of in vivo‐jetPEI into 10 µL of 10% glucose, adding sterile water to 20 µL, vortex gently and spin down. Next, add the diluted in vivo‐jetPEI to the diluted siRNA or mRNA at once, vortex briefly and spin down. Incubate for 15 min at room temperature. Perform injections into the ovary using a thin glass needle with a mouthpiece. For each injection, the optimal volume was ≈0.3 µL per ovary at room temperature. The CON group was infected with NC‐siRNA or in vivo‐jetPEI transfection reagent.

**Table 1:** List of siRNAs.

Gene	Forwards sense	Antisense	Label
*NC*	UUCUUCGAACGUGUCACGUTT	ACGUGACACGUUCGGAGAATT	5’ 6‐FAM
*Arpc4*	GAGCAGAGAACUUVUUUAUTT	AUAAAGAAGUUCUCUGCUCTT	5’ 6‐FAM
*Myosin IIA*	GUAAAUUCAUUCGUAUCAATT	UUGAUACGAAUGAAUUUACCG	5’ 6‐FAM
*Myosin IIB*	GCUACUAUUCAGGACUUAUTT	AUAAGUCCUGAAUAGUAGCGG	5’ 6‐FAM
*Myosin IIC*	CGGAGGCCAUUGUUGAAAUTT	AUUUCAACAAUGGCCUCCGTT	5’ 6‐FAM

**Table 2:** List of primers used for quantitative real‐time PCR.

Genes		Forwards (5′ to 3′)	Reverses (5′ to 3′)	
*Myosin IIA*	GGCCCTGCTAGATGAGGAGT		CTTGGGCTTCTGGAACTTGG
*Myosin IIB*	GGAATCCTTTGGAAATGCGAAGA		GCCCCAACAATATAGCCAGTTAC
*Myosin IIC*	CAGTGACCATGTCCGTGTCTG		CGTAGAGGAACGATTGGGCTG
*Gdf9*	TCTTAGTAGCCTTAGCTCTCAGG		TGTCAGTCCCATCTACAGGCA
*Bmp15*	TCCTTGCTGACGACCCTACAT		TACCTCAGGGGATAGCCTTGG
*Nobox*	AAGACCCGAACCCTGTACC		CTCATGGCGTTTGTCACTGTC
*Filia*	TGCCCCTGGATCACAAACAAG		GAGGTTCCACGTACAGTCTTTTT
*Lhx8*	TCAGAGAGTGGTTACGGTCAC		CTGCTCGTCACATACCAGCTC
*Notch*	ATGTGGACGAGTGTCTGTTGC		GGAAGCATAGGCACAGTCATC
*Hey2*	AAGCGCCCTTGTGAGGAAAC		GGTAGTTGTCGGTGAATTGGAC
*Tle6*	ATCCAGTCGGTATTTGTCCATCG		AGGTCTGGGGTTCTACTGAAG
*Nlrp*	GAAAGCACAATGGGTCCTCCA		CTGACGCCTGTTCCACTTCT
*mtDNA*	TACCTCACCATCTCTTGCTA		CCACATAGACGAGTTGATTC
*β‐Globin*	CGAACATACTGAACTGCTA		GACATATCTGACATCTCTACTT
*DDX4*	GCTTCATCAGATATTGGCGAGT		GCTTGGAAAACCCTCTGCTT

### Proteomics Sequence Analysis

Proteomics sequencing services were provided by Jingjie Biotechnology Co., Ltd. (Hangzhou, China). E16.5 ovaries were cultured in CON and CB ovarian culture media for 2 days, followed by protein extraction, trypsin digestion, TMT/iTRAQ labelling, high‐performance liquid chromatography (HPLC) fractionation, mass spectrometry analysis, database search, bioinformatics analysis.

### Immunofluorescence Staining

Ovary frozen section immunofluorescence staining: ovaries were fixed in 10% neutral formalin fixative for at least 12 h; ovaries were then embedded in Optimal Cutting Temperature Compound (OTC) after dehydration in 15% sucrose‐PBS for 4 h and 30% sucrose‐PBS for 2 h. Ovarian tissues were serially sectioned at 8 µm under a freezing microtome (Tissue‐tek, POLAR D). After dewaxing, rehydration and 96–98 °C antigen retrieval with 50X Sodium Citrate Antigen Retrieval Solution (C1032, Solarbio), the sections were blocked with 10% FBS‐PBS for 2 h at room temperature (RT) and immunostained with primary antibodies at 4 °C overnight. Slides were then washed with PBST (PBS with 0.1% Tween 20). Subsequently, the sections were incubated with secondary antibodies at RT for 3 h and washed with PBST. In the end, sections were incubated with DAPI at RT for 15 min. Following the staining process, slides were then rinsed in PBST and sealed in anti‐fade fluorescence mounting medium (ab104135, Abcam) with coverslips. Sections were examined and photographed by a confocal microscope (Leica TCS SP8).

### Oocyte Immunofluorescence Staining

Oocytes were fixed with 4% paraformaldehyde for 30 min, followed by permeabilization with 1% Triton X‐100 in PBS for 1 h. They were further blocked with 1% BSA‐PBS for 1 h at RT. Next, the oocytes were incubated with primary antibodies at 4 °C overnight, then washed 3 times with PBST. Subsequently, the oocytes were incubated with secondary antibodies at RT for 3 h and washed three times with PBST. In the end, oocytes were incubated with DAPI at RT for 15 min. Following the staining process, the samples were transfered to glass slides and subsequently detected by a confocal microscope (Leica TCS SP8).

### RNA Isolation and Real‐Time Quantitative PCR Analysis

TRIZOL Reagent (T9424, Sigma) was used to extract ovarian total RNA. cDNA was synthetized by Evo M‐MLV RT Mix Kit (AG11705, ACCURATE BIOLOGY). The primer sequences for the respective genes are listed in Table 1. Real‐time PCR reaction system (10 µL) consisted of 2x SYBR Green Pro Taq HS 5 µL; forward primer 0.2 µL, reverse primer 0.2 µL; cDNA and ddH20 4.6 µL. Real‐time PCR was conducted with a real‐time PCR system (ABI Step One Plus). The 2^−ΔΔCt^ method was applied to analyze the relative gene expression levels.

### Western Blot Analysis

Each protein sample was extracted in RIPA Lysis Buffer [36 P0013K, Beyotime] containing 1% PMSF. The protein samples were then subjected to a heating process at 100 °C for 10 min. The proteins were then separated through SDS‐PAGE, with an initial voltage of 80 V for 30 min, followed by 100 V for 90 min. Upon completion of electrophoresis, the protein was transferred to the PVDF membrane (Millipore, Billerica, MA, USA) through the application of 50 V for 120 min. Following the transfer process, the membranes were subjected to blocking with 3% non‐fat milk dissolved in TBST (TBS+0.1% Tween 20) at RT for 90 min. After the blocking procedure, the membranes were incubated with primary antibodies at 4 °C overnight; after that, cave rounds of washing the PVDF membranes in TBST, with each washed 5 min. Then, expose the membranes to Donkey anti‐rabbit IgG or Donkey anti‐mouse IgG at RT for 1 h. Following a series of 5 rinses, the membranes were subjected to visualization via the ECL ENhanced Kit (DiNing, Cat# DE2001‐100).

### Statistical Analysis

For every analysis, a minimum of three biological replicates were performed, and the results were presented as means ± SEM. Sample size (n) for each statistical analysis is indicated in the corresponding Figure or Figure legends. Data were analyzed by t‐test or one‐way ANOVA. The software Imaris was employed to calculate organelle volume, while all statistical analyses were conducted using GraphPad Prism 7.00 software (GraphPad, CA, USA). *p* < 0.05 represented by ^*^ and different letters; *p* < 0.01 represented by ^**^; *p* < 0.001 represented by ^***^; and *p* < 0.0001 represented by ^****^.

### Ethics Statement

All experiments in this research were complied with the principles and guidelines for the use of laboratory animals and approval by the Institutional Animal Care and Use Committee of College of Veterinary Medicine, Northwest A&F University (No.2021070712).

## Conflict of Interest

The authors declare no conflict of interest.

## Supporting information



Supporting Information

Supplemental Movie 1

Supplemental Movie 2

Supplemental Movie 3

Supplemental Movie 4

Supplemental Movie 5

Supplemental Movie 6

Supplemental Movie 7

## Data Availability

The data that support the findings of this study are available from the corresponding author upon reasonable request.
