# Supplementary figures and images for: Microfilament‐Myosin II Regulates the Differentiation of Multinucleated Cysts into Oocytes and Influences Oocyte Developmental Potential in Mice
Source: Adv Sci (Weinh). 2025 Sep 17;12(45):e00358. doi: 10.1002/advs.202500358 (PMC12677636; doi:10.1002/advs.202500358)

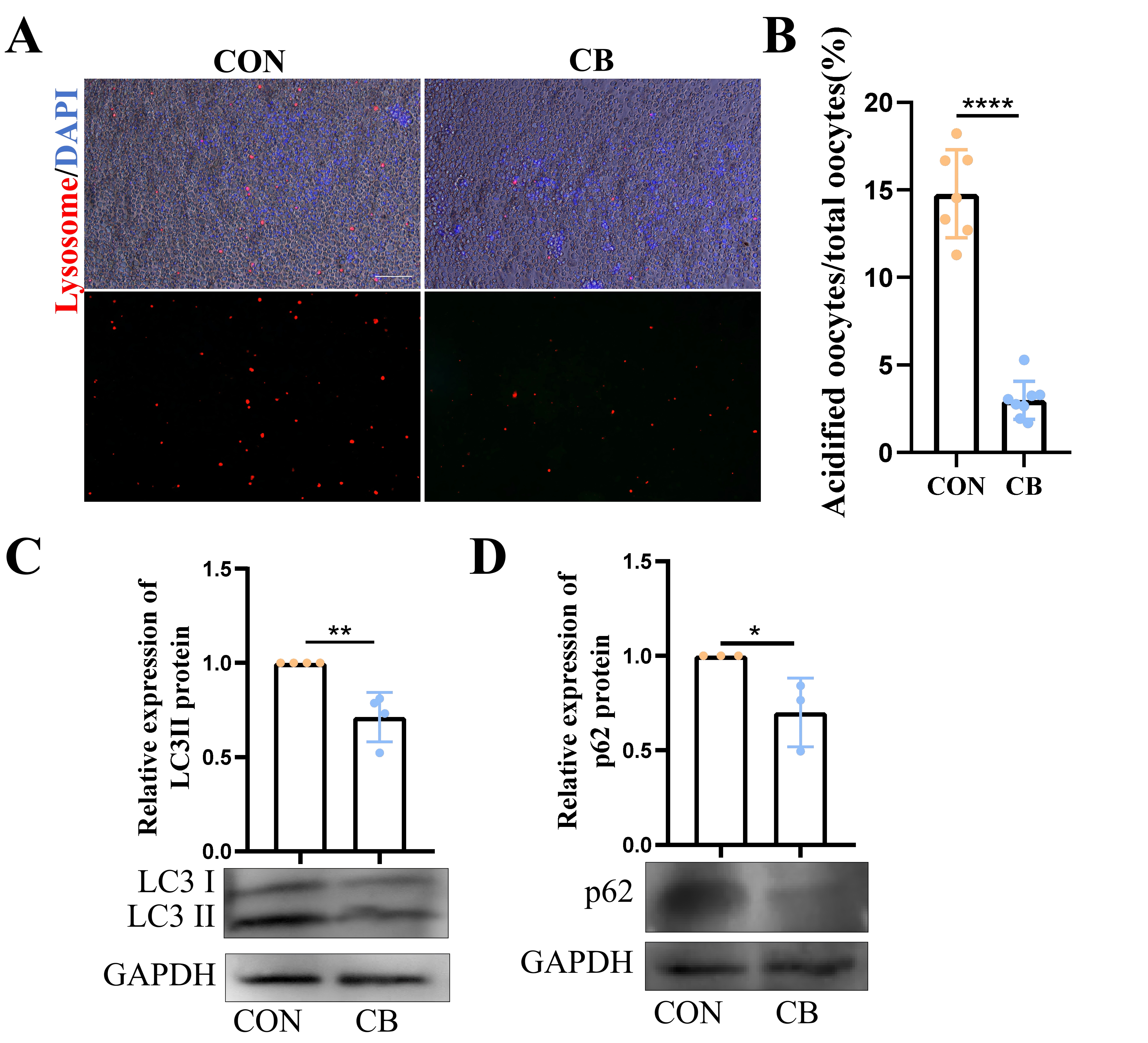

Supplement: Supplementary file 1 — Supporting Information [file ADVS-12-e00358-s001.zip › Figure S2.jpg]

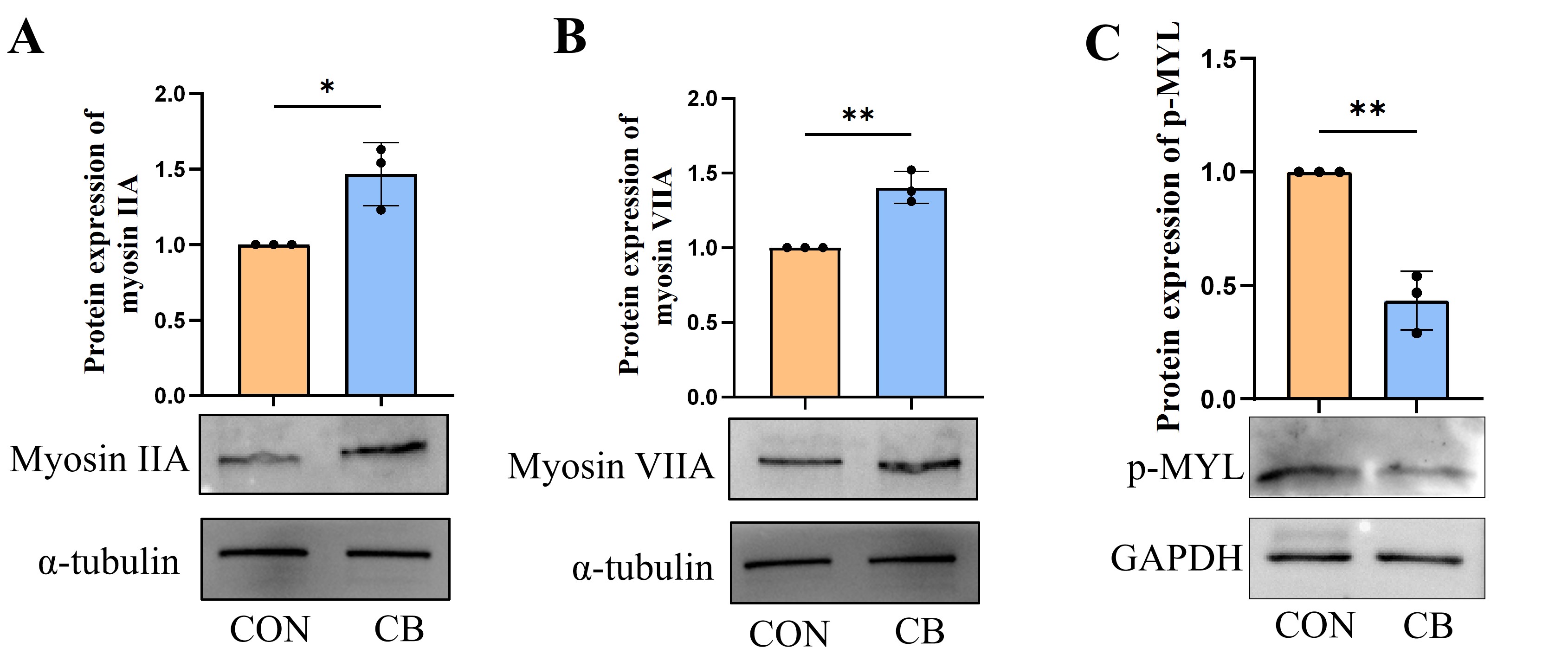

Supplement: Supplementary file 1 — Supporting Information [file ADVS-12-e00358-s001.zip › Figure S3.jpg]

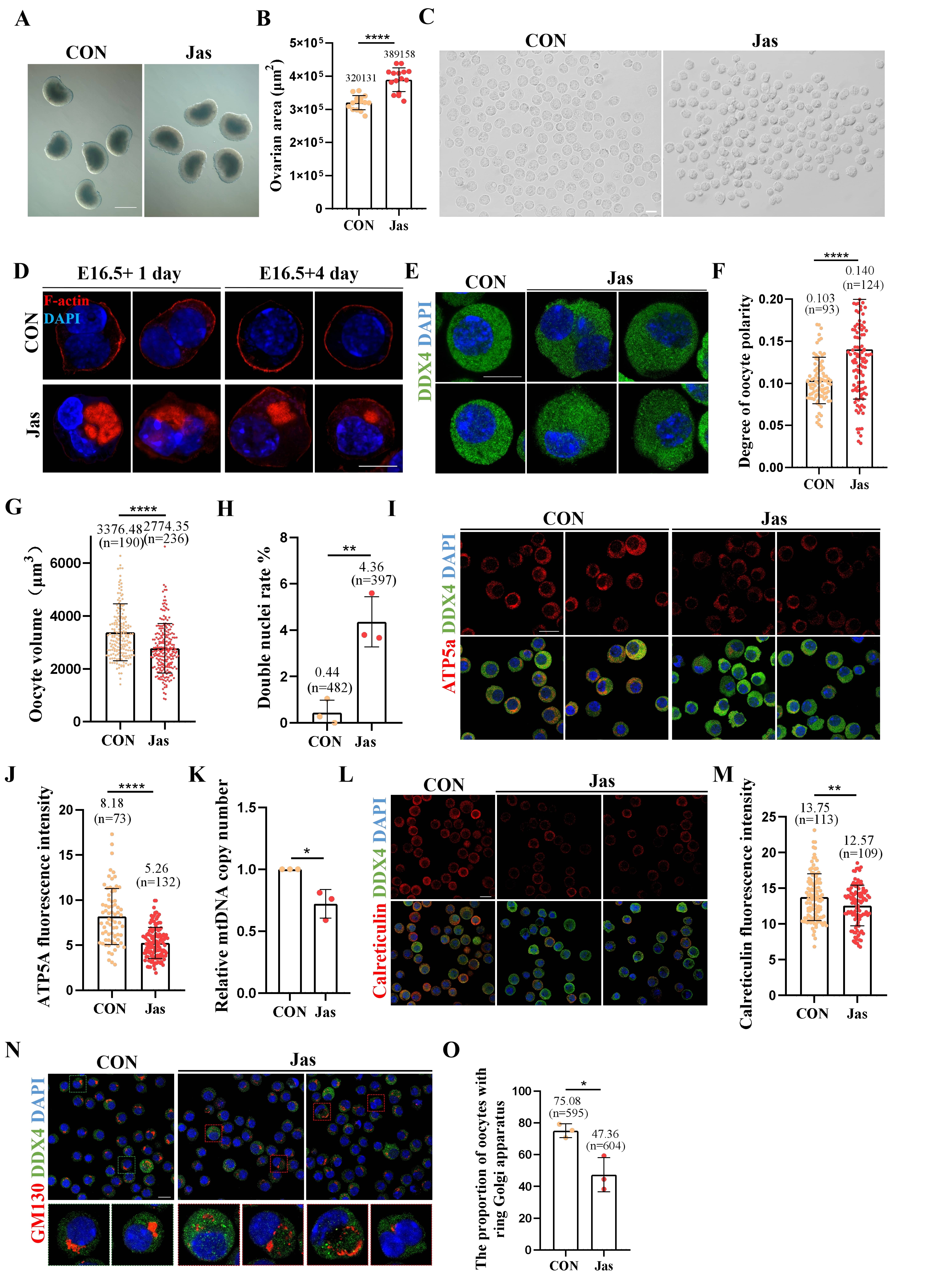

Supplement: Supplementary file 1 — Supporting Information [file ADVS-12-e00358-s001.zip › Figure S4.jpg]

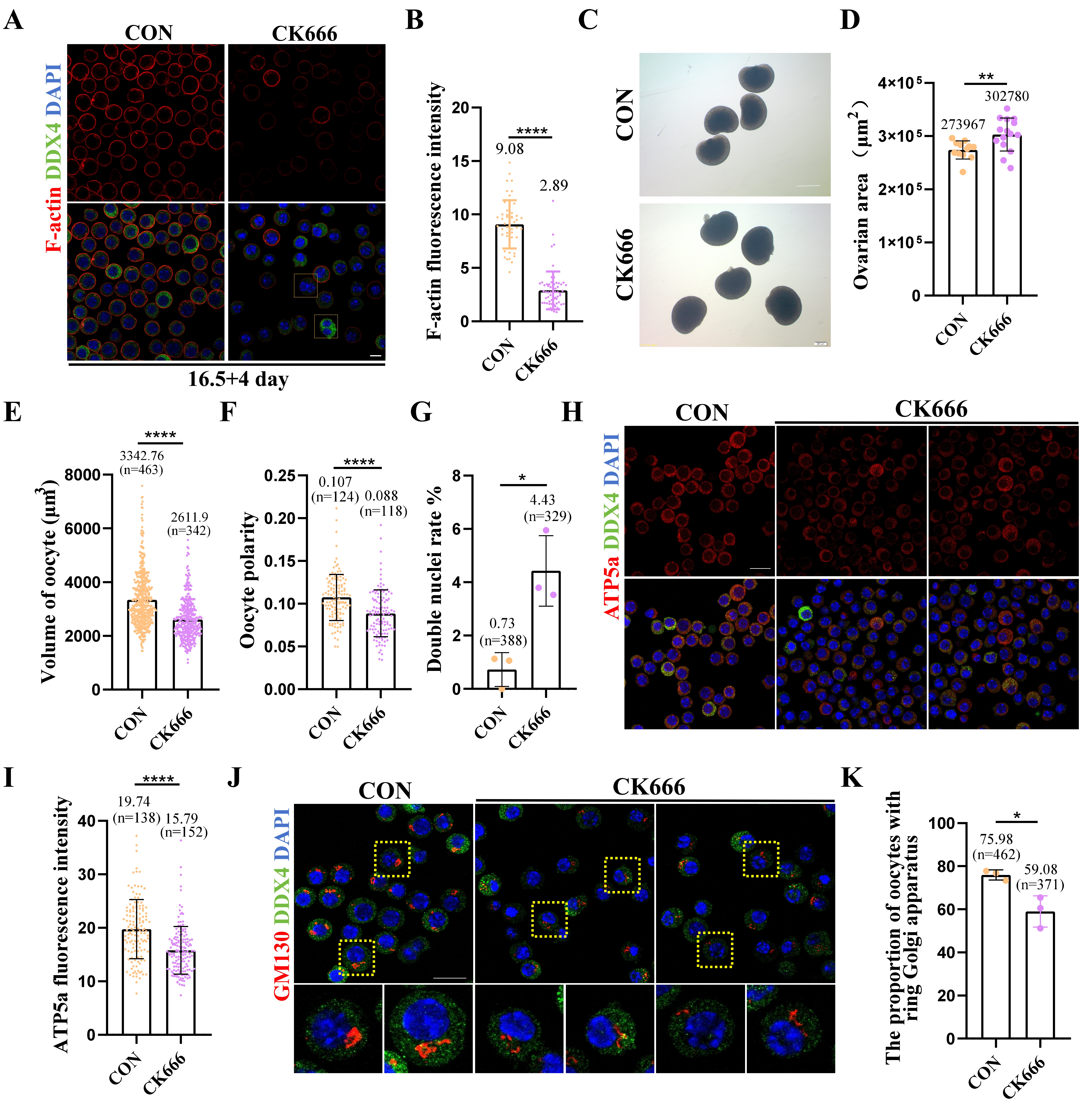

Supplement: Supplementary file 1 — Supporting Information [file ADVS-12-e00358-s001.zip › Figure S5.jpg]

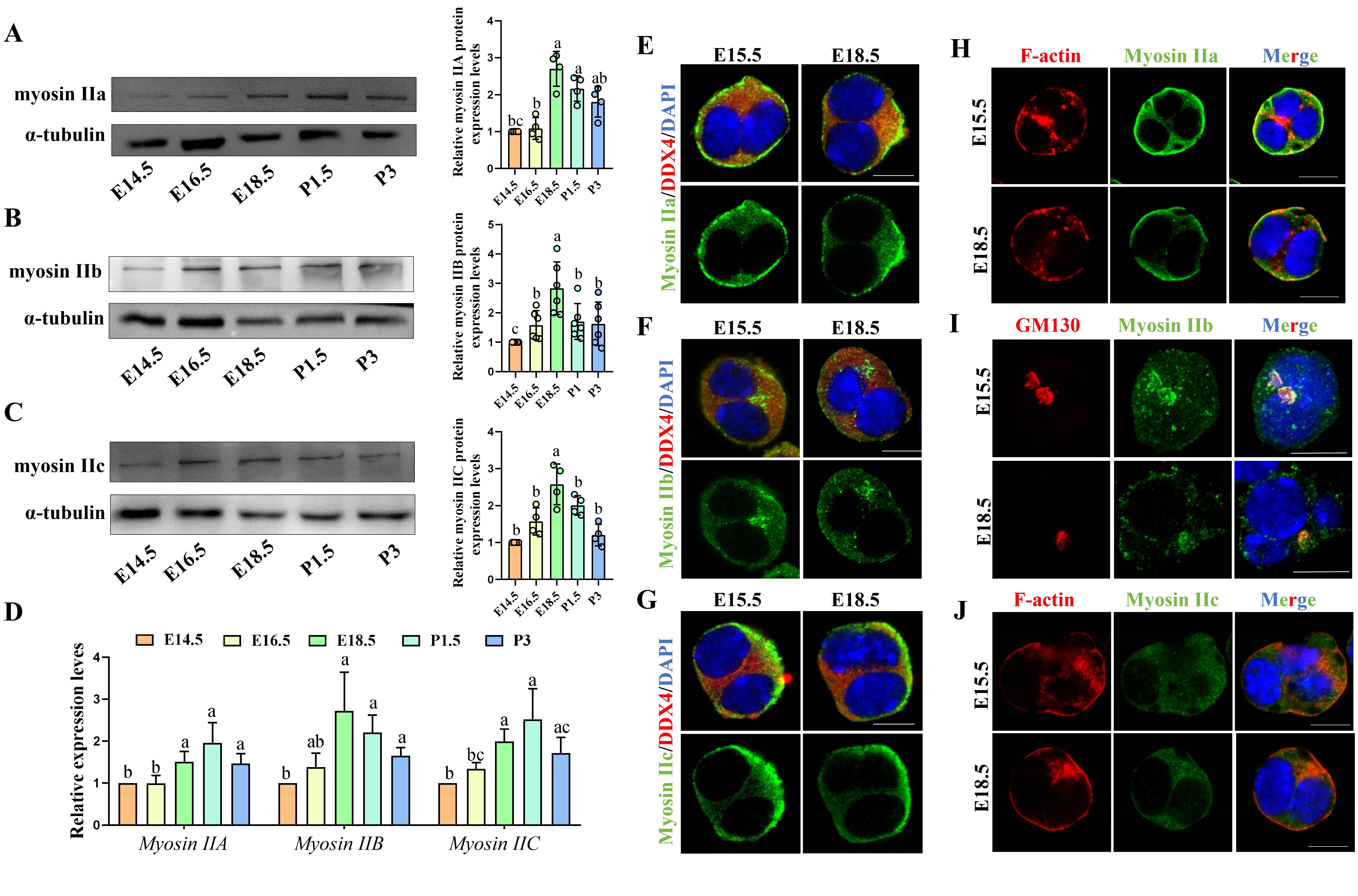

Supplement: Supplementary file 1 — Supporting Information [file ADVS-12-e00358-s001.zip › Figure S6.jpg]

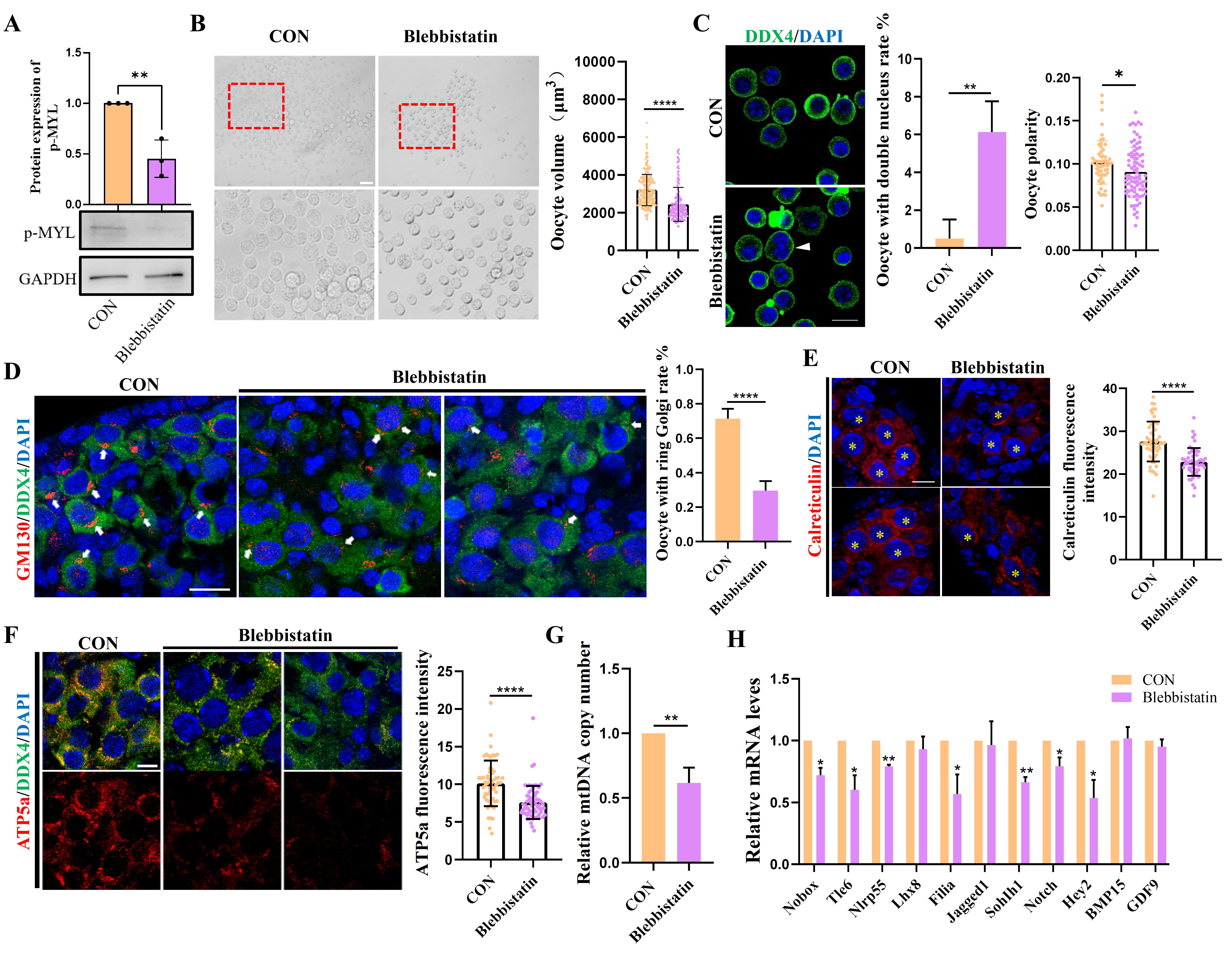

Supplement: Supplementary file 1 — Supporting Information [file ADVS-12-e00358-s001.zip › Figure S7.jpg]

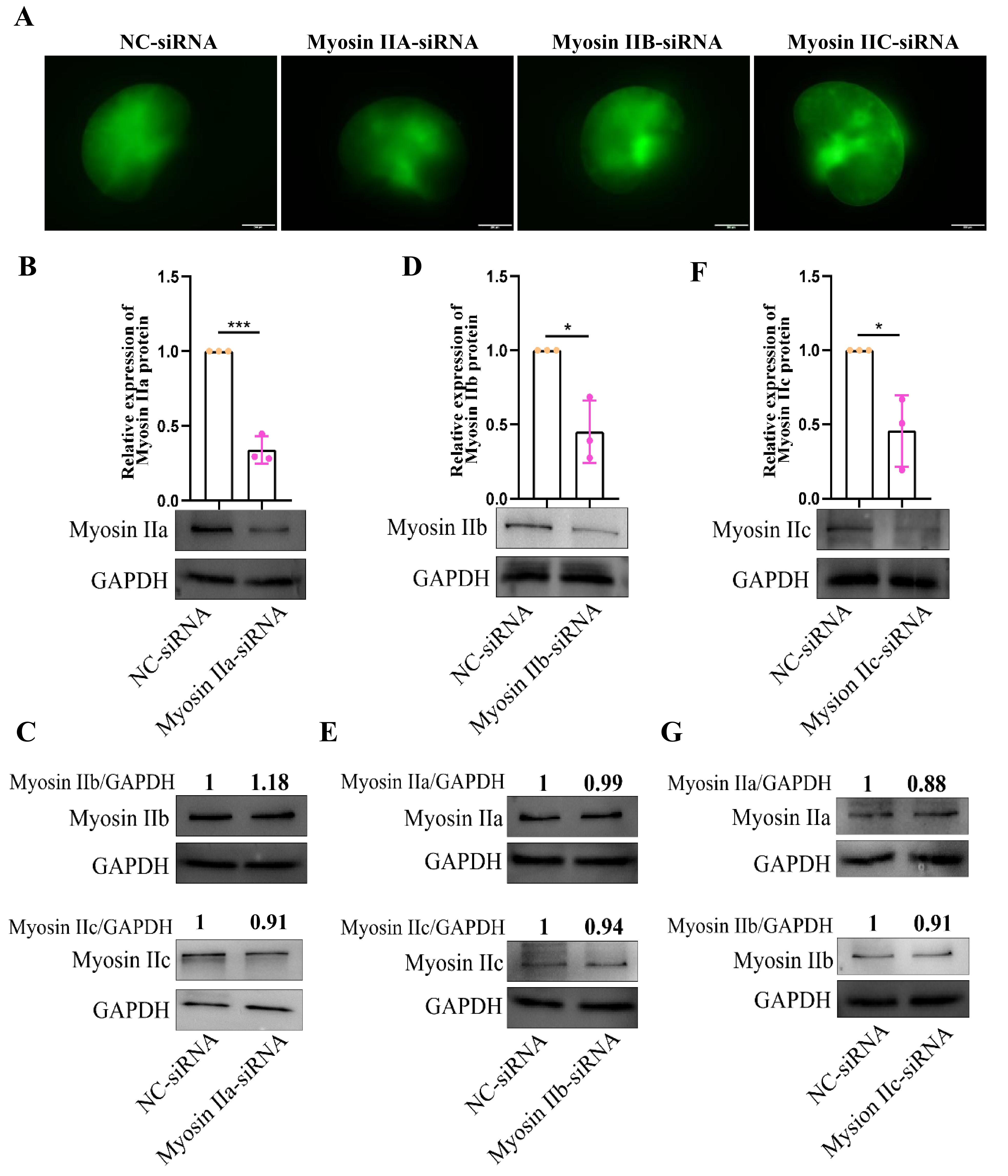

Supplement: Supplementary file 1 — Supporting Information [file ADVS-12-e00358-s001.zip › Figure S8.jpg]

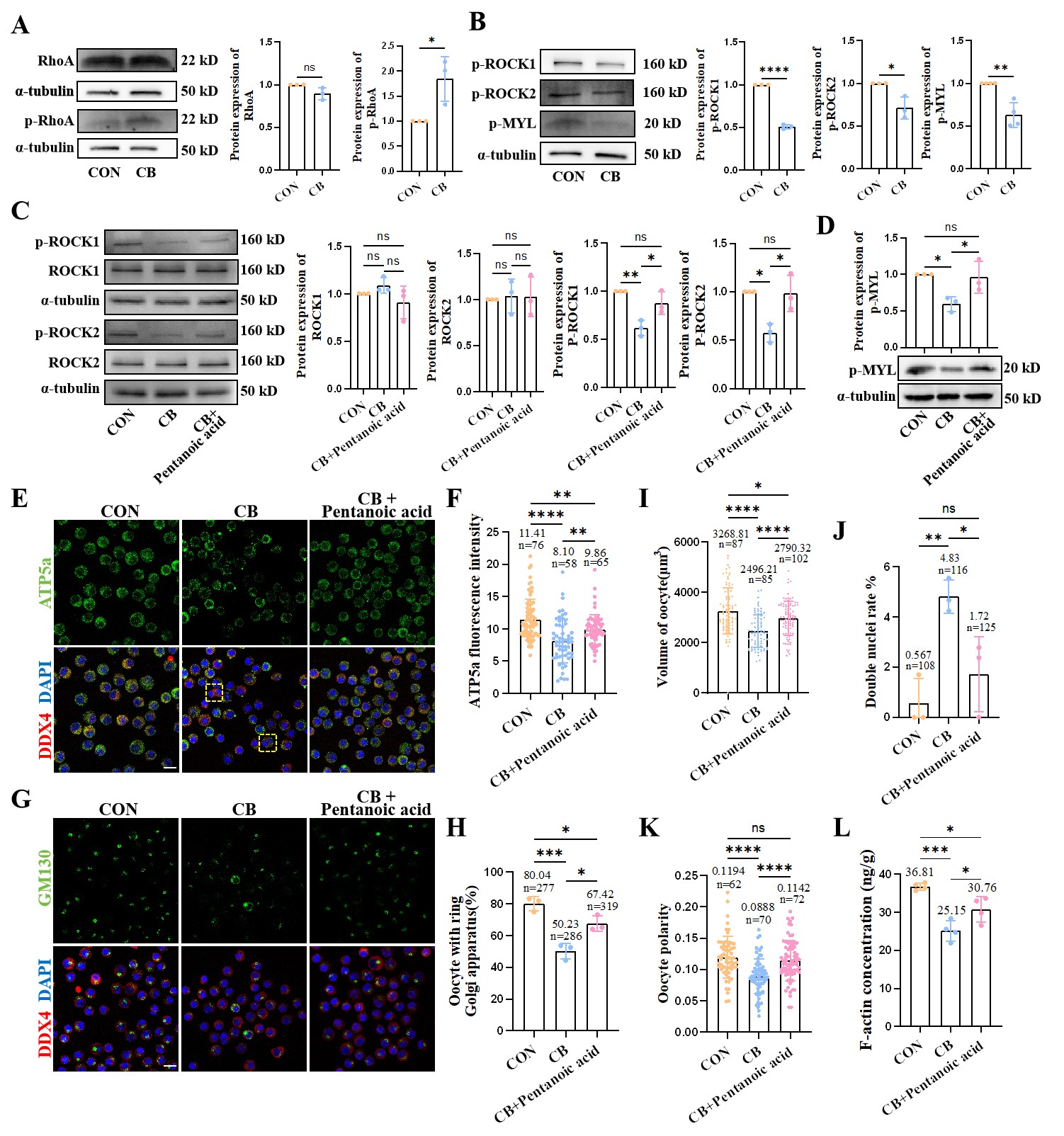

Supplement: Supplementary file 1 — Supporting Information [file ADVS-12-e00358-s001.zip › Figure S9.jpg]

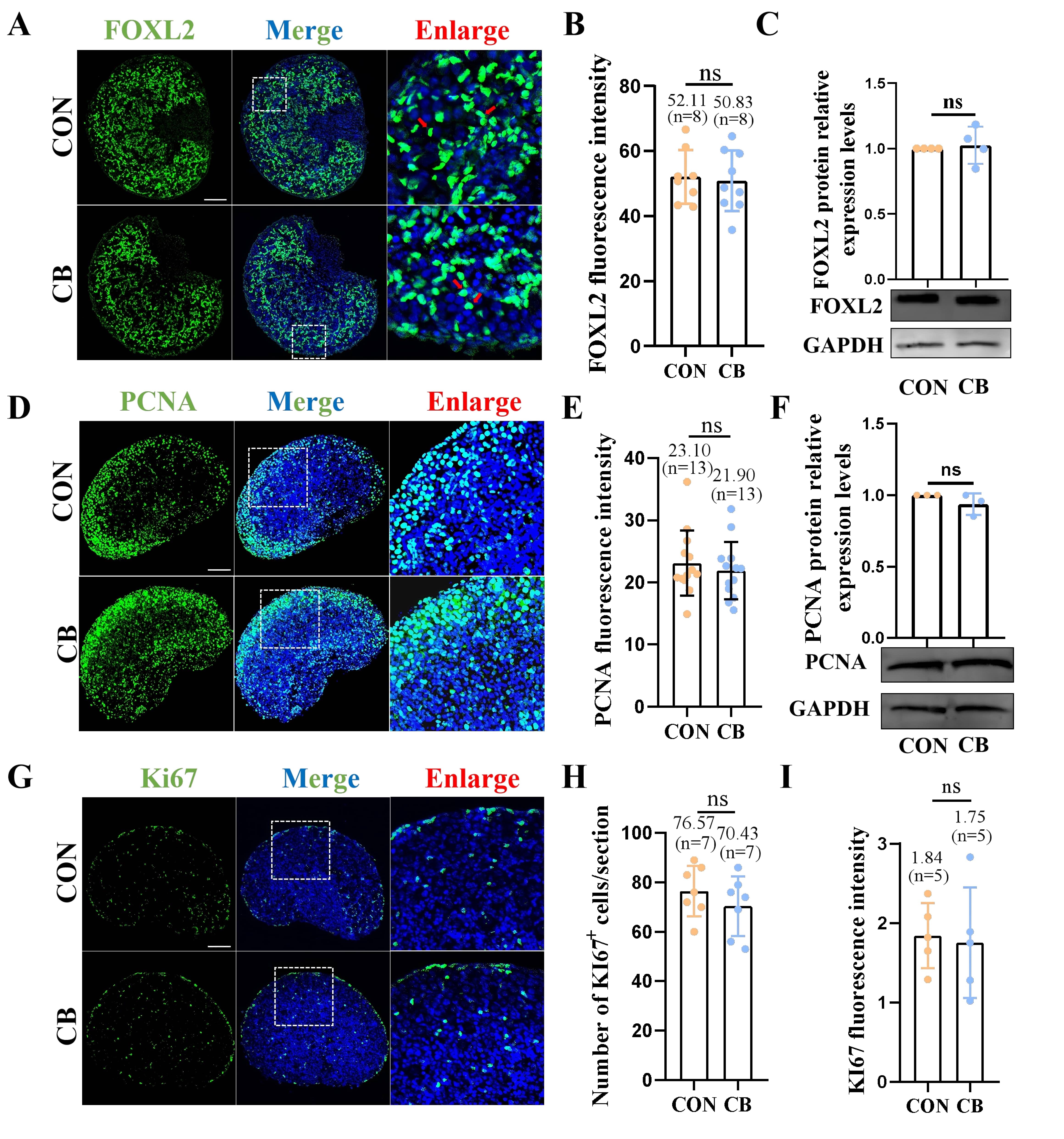

Supplement: Supplementary file 1 — Supporting Information [file ADVS-12-e00358-s001.zip › Figure S10.jpg]

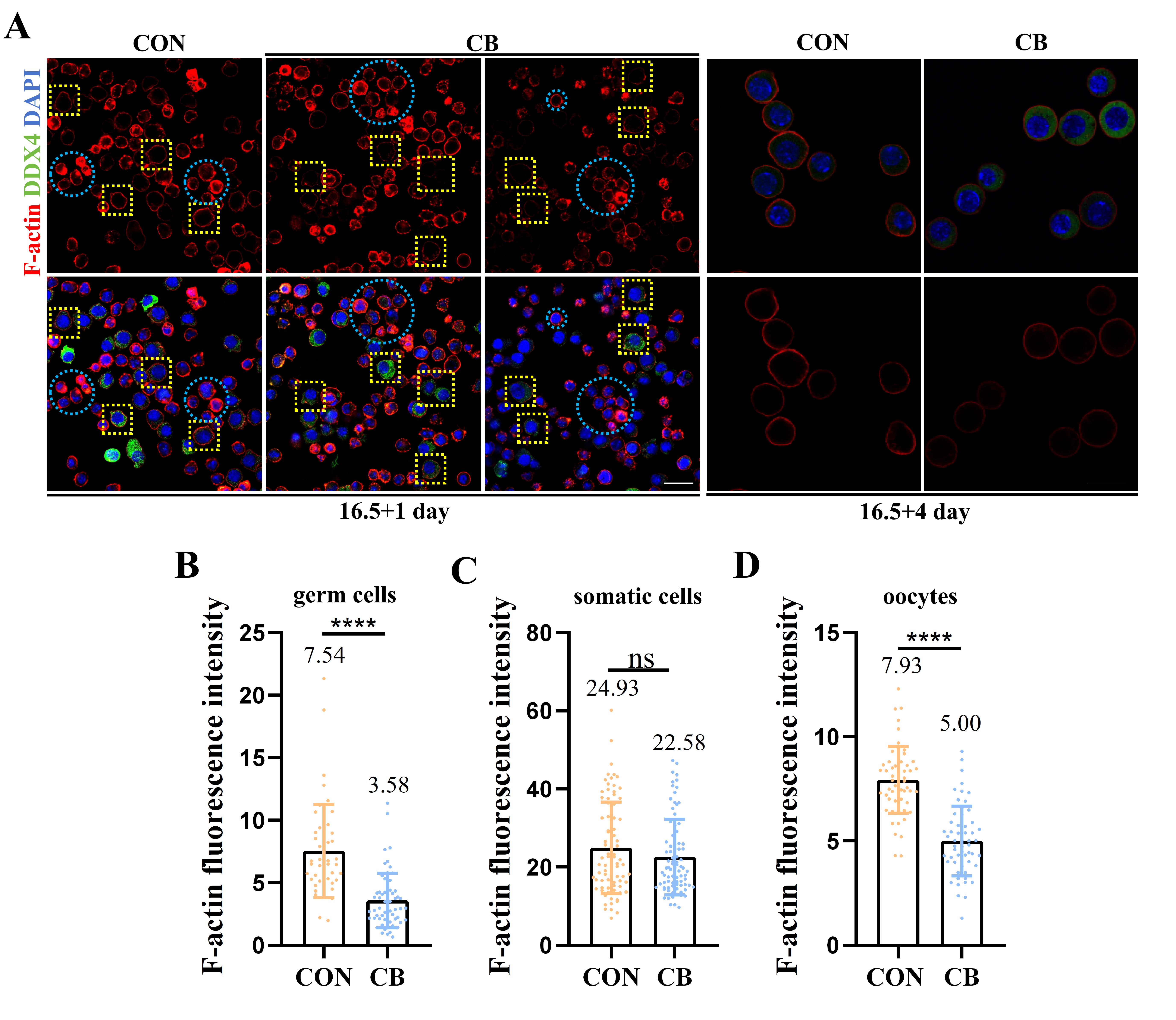

Supplement: Supplementary file 1 — Supporting Information [file ADVS-12-e00358-s001.zip › Figure S1.jpg]
